# New Anticancer 4‐Aryldihydropyrimidinone‐5‐Carboxylates Targeting Hsp90

**DOI:** 10.1111/cbdd.70221

**Published:** 2025-12-15

**Authors:** Cinzia Bordoni, Tia Z. Elstow, Andrea Brancale, Richard W. E. Clarkson, Andrew D. Westwell

**Affiliations:** ^1^ School of Pharmacy and Pharmaceutical Sciences Cardiff University Cardiff Wales UK; ^2^ European Cancer Stem Cell Research Institute, School of Biosciences Cardiff University Cardiff Wales UK; ^3^ Department of Organic Chemistry University of Chemistry and Technology Prague Czech Republic

**Keywords:** anticancer drug design, breast cancer, Hsp90 inhibition, multicomponent Biginelli reaction

## Abstract

Amplified expression of the heat shock protein 90 (Hsp90) chaperone within cancer cells is associated with poor patient prognosis, and is known to drive tumour invasion, treatment resistance and metastasis within a range of tumour types. Several Hsp90 small molecule inhibitors have progressed into clinical development; however, progress in the clinic to date has been sub‐optimal, and new Hsp90 inhibitor chemical scaffolds are needed to realise the full potential of this target. Following identification of a virtual hit compound able to mimic interactions of the Hsp90 inhibitor geldanamycin within the Hsp90 ATP binding site, we have designed and synthesised a range of substituted 4‐aryldihydropyrimidinone‐5‐carboxylate derivatives (**5a–n**) for structure–activity relationship (SAR) study as Hsp90 inhibitors within human breast cancer cell models. Compound **5d** emerged as the most promising analogue, combining low levels of growth inhibition with potent inhibition of colony formation in breast cancer cell lines, and effective Hsp90 inhibition. Early ADME profiling confirmed **5d** to have moderate metabolic stability and solubility, and to lack hERG channel inhibition, confirming **5d** as a useful hit compound for further development.

## Graphical abstract

We report the computational design and early structure–activity relationship optimisation (SAR) studies on new anticancer 4‐aryldihydropyrimidinone‐5‐carboxylate derivatives as inhibitors of heat shock protein 90. Analogue **5d** emerged as a useful hit compound, combining inhibition of colony formation in breast cancer cell lines with effective Hsp90 inhibition and promising ADME properties.

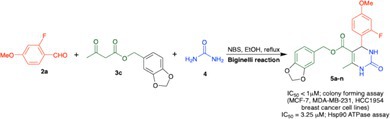

## 1 Introduction

The heat shock protein 90 (Hsp90) chaperone plays a pivotal role in the folding, stabilisation and activity of client oncoproteins, preventing proteasomal degradation (Grammatikakis et al. 2002). Amplified expression of heat shock proteins such as Hsp90 within cancer cells is associated with poor prognosis, tumour invasion, treatment resistance and metastasis in a range of human cancer types (Rastogi et al. 2024).

The Hsp90 family exists as functional paralogs in mammalian cells, with the stress‐induced cytosolic and nuclear forms known as Hsp90α (HSP90AA1) and Hsp90β (HSP90AB1) most widely implicated in various solid tumours such as lung, hepatocellular and colorectal cancer (Rastogi et al. 2024). Hsp90 exists as a homodimer composed of three different domains: an *N*‐terminal nucleotide‐binding domain (NTD) with ATPase activity; a central domain for client protein binding; and a *C*‐terminal domain involved in dimerization (Picard 2002). Numerous cancer drug discovery projects have focused on the design of Hsp90 inhibitors, mainly targeting the *N*‐terminal domain, inspired by the benzoquinone‐containing natural product geldanamycin and synthetic derivatives (Kitson et al. 2024).

**FIGURE 1:**
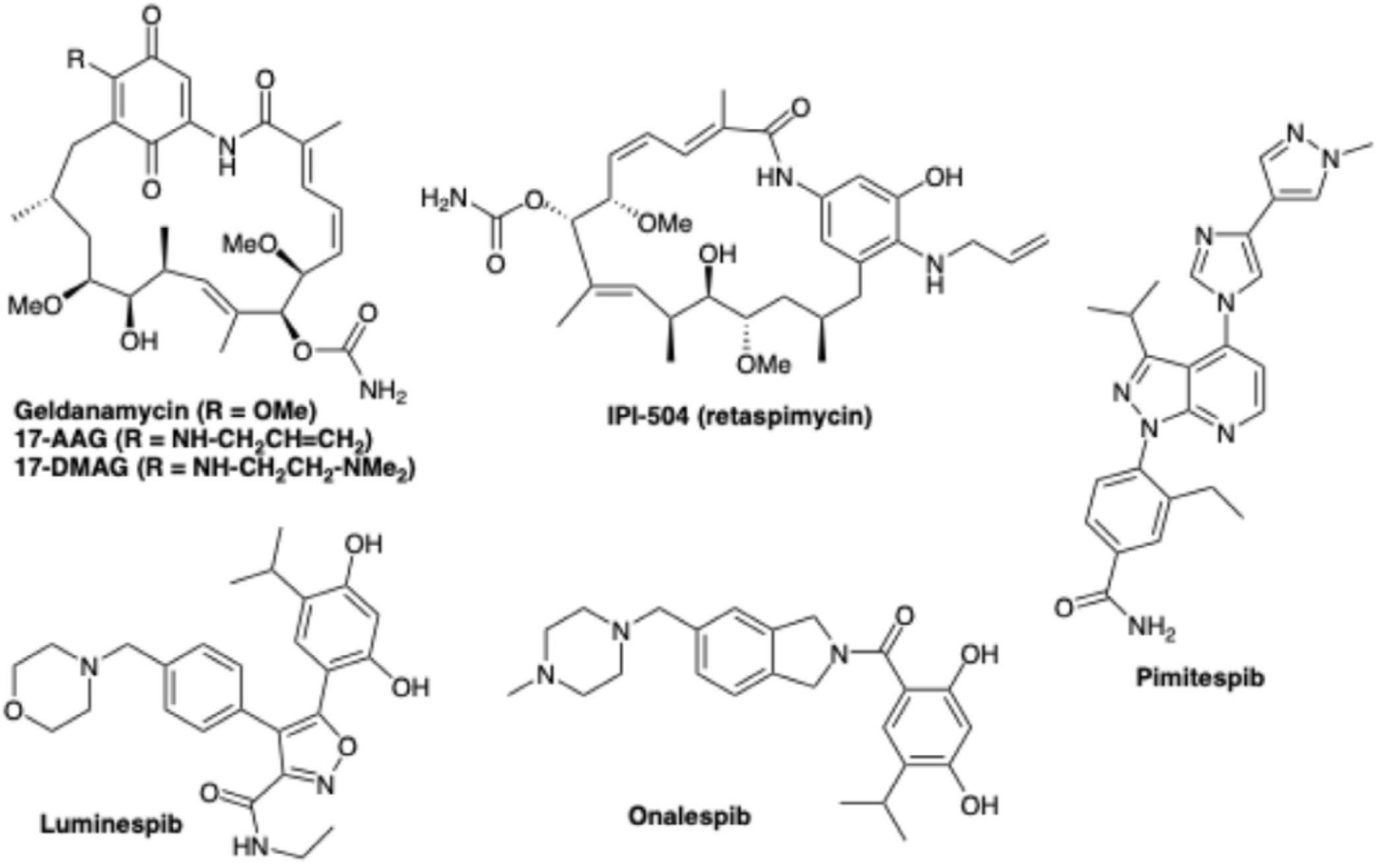
Structures of representative clinical stage Hsp90 inhibitors.

Interestingly, countering the concerns due to the housekeeping function of Hsp90, it was found that ATP binds in a bent conformation in the *N*‐terminal domain. This unique feature of the ATP‐binding pocket may present a solution to selective therapeutic targeting of the Hsp90 ATPase domain. Consequently, several small drug‐like molecules targeting the ATPase domain were progressed to Phase I and II cancer clinical trials (Rastogi et al. 2024); selected examples are shown in Figure 1. Important early examples include the natural product geldanamycin and its synthetic derivatives, such as 17‐AAG and 17‐DMAG, which were ultimately found to exhibit sub‐optimal solubility, bioavailability and off‐site toxicities. IPI504 was characterised by an improved solubility, pre‐clinical activity and toxicity profile, but showed a sub‐optimal clinical response. Luminespeb was tested in clinical trials in 2011, but progression halted in 2014. Onalespib (Astex) is currently under evaluation in several Phase I and II clinical trials (Shrestha et al. 2016; Tatokoro et al. 2015). Notably, the Hsp90 inhibitor pimitespib (Taiho Pharmaceutical Co. Ltd.) received approval in Japan in 2022 for the treatment of gastrointestinal stromal tumour (GIST) that has progressed after chemotherapy (Doi et al. 2024). Although initially regarded with some skepticism as a druggable target in anticancer drug discovery, over the last two decades the pharmaceutical industry has developed a growing interest in designing Hsp90 inhibitors (Kadasi et al. 2018). To date, more than a dozen different clinical candidates have progressed to clinical trials (Liu et al. 2024). Limitations around the pharmacokinetic properties of the clinical candidate targeting the Hsp90 signaling pathway have tended to be the common drawback associated with the earlier development candidates. However, Hsp90 continues to represent an interesting cancer drug target, and further novel lead molecules are needed to identify non‐cytotoxic Hsp90 inhibitors for the benefit of cancer patients, especially within combination therapy regimes alongside chemotherapy and/or targeted therapy.

**FIGURE 2:**
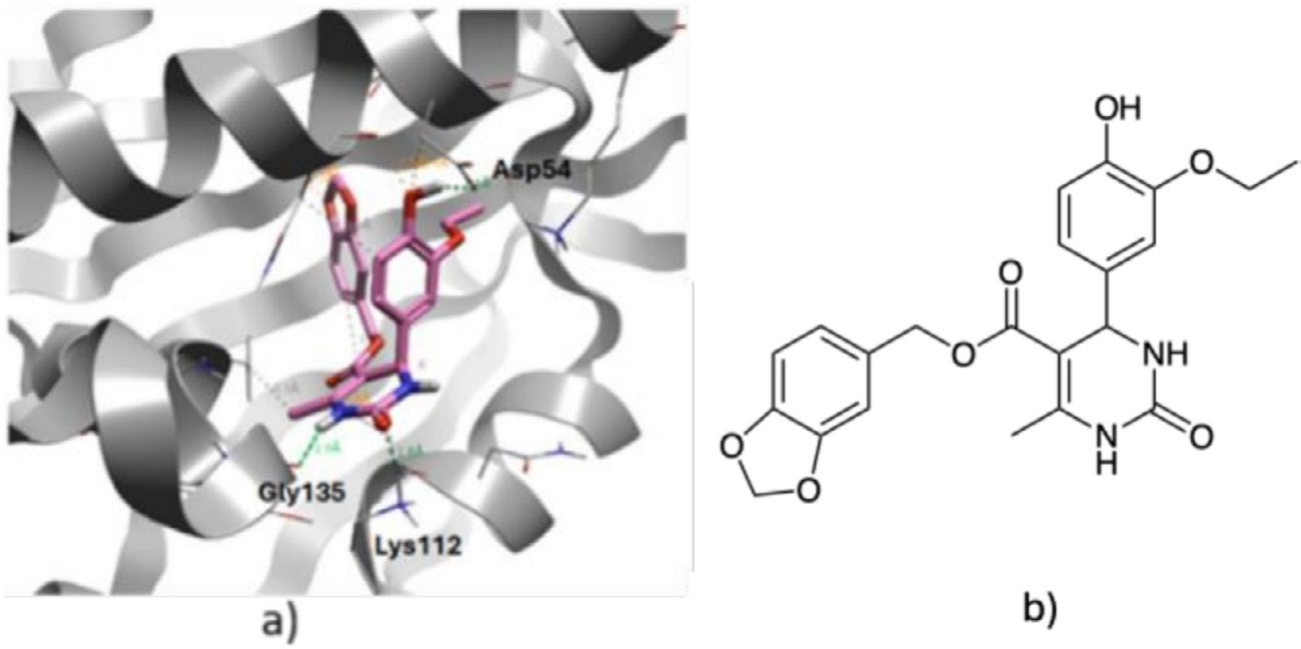
(a) Docking pose of compound **1** in the ATP binding site of Hsp90 (PDB code: 1YET), showing key interactions with Asp 54, Lys112 and Gly 135. (b) Chemical structure of compound **1** (AG‐205/37136062, Specs database).

In related work within our group towards novel anti‐metastatic Bcl3 inhibitor drug candidates, a high throughput virtual screen against a new Bcl3 binding pocket led to 10 putative hit compounds from the SPECS database with distinct pharmacophores (Soukupová 2013). Further in vitro evaluation of the Bcl3‐p50 protein–protein binding inhibition led to a new anthranilic acid derivative (known as JS6) being progressed for further development (Soukupová et al. 2021; Westwell et al. 2015). Of the remaining virtual hit series, a 4‐arylpyrimidone‐5‐carboxylate derivative (**1**, AG‐205/37136062; Figure 2) stood out (Soukupová 2013). Whilst not active within the Bcl3‐binding assays, compound (**1**) exhibited potent inhibitory activity in our colony forming assay within the invasive breast cancer cell line HCC1954 (Bordoni 2016). Other notable features of compound **1** included previous reports of a similar chemical scaffold with activity against Hsp90 (Terracciano et al. 2016). Further, assessment of (**1**) in a reverse docking approach using the Drug Repositioning, Adverse Reaction via Chemical‐Protein Interactome (DRAR‐CPI) server (Elixir Bio.tools; https://cpi.bio‐x.cn/drar/) (Luo et al. 2011), and SwissTargetPrediction (SwissDrugDesign 2011), Hsp90 was identified as a likely molecular target.

In this paper, we describe the structure‐based design, chemical synthesis and in vitro anticancer evaluation of 4‐arylpyrimidinone‐5‐carboxylate analogues (**5a–n**) for structure–activity relationship (SAR) studies within a range of cellular models. We identify analogue (**5d**) as a drug‐like Hsp90 inhibitory compound with only minor effects on cancer cell viability, but with profound impacts on breast cancer cell colony formation.

## 2 Materials and Methods

### 2.1 Molecular Modelling

All molecular modelling studies were performed on a MacPro 2.66 GHz Quad‐Core Intel Xeon, running Ubuntu. Molecular Operating Environment (MOE) 2013.08 and Maestro (Schrodinger version) were used as molecular modelling software. All minimisations were performed with MOE until RMSD gradient of 0.001 kcal mol^−1^ Å^−1^ with AMBER99 force field. Partial charges were automatically calculated. Conformational analyses were performed with MOE 2013.08. Docking experiments were carried out using LeadIT‐FlexX version 2.10, PLANTS version 1.1, and the GlideSP module in Maestro with the default options.

### 2.2 General Chemical Methods

All commercially available reagents were purchased without further purification. Compound 1 (AG‐205/37136062) was purchased from the Specs database (specs.net). Compound 1 was submitted to the publicly available filter to confirm the likely absence of PAINS (Pan Assay INterference compoundS) (Baell and Walters 2014). Reactions were carried out under nitrogen in oven‐dried glassware. Reactions were monitored by thin‐layer chromatography (TLC) using silica gel plates (Merck Kieselgel 60F254) and visualised under UV light at 254 and 366 nm. Microwave‐promoted reactions were conducted in a 10 mL glass vessel in the microwave cavity of the Discover Labmate CEM microwave reactor in closed vessel mode. Flash column chromatography was carried out using silica gel 40–60 μm (Merck) or using the Interchim PuriFlash 4000 automated column chromatography system. ^1^H‐NMR, ^13^C‐NMR, ^19^F‐NMR spectra were recorded using a Bruker AVANCE (500, 125 and 202 MHz) spectrometer, with TMS used as an internal standard. Chemical shifts (*δ*) are in ppm relative to tetramethylsilane together with the relative assignment (Ar = aryl), the coupling constant (*J*) in Hz, and the multiplicity: singlet (s), doublet (d), triplet (t), quartet (q), multiplet (m). Low‐resolution mass spectra were performed on Bruker Daltonics microTof‐LC in positive or negative mode electron spray ionisation mass spectroscopy (ESI). Purity of tested compounds was > 95%, as estimated by analytical high‐performance liquid chromatography (HPLC) experiments. These were carried out on a Series 200 UV/Vis Detector provided with a System Controller SN4000, and a Spectra System P4000 pump with flow range of 0.1–10.0 mL/min; and a Series 200 UV/Vis Detector (at 254 and 280 nm) using a C18‐Varian Pursuit (150 × 4.6 mm, 5 μM) reverse phase column. The reactions were monitored by HPLC using the eluents water (eluent A) and methanol (eluent B), under the following conditions: gradient from 100% → 70% of eluent A in 15 min, then to 100% of eluent B in 15 min (method 1). Purity of tested compounds was also estimated by analytical ultra‐performance liquid chromatography (UPLC) experiments on an Acquity UPLC H‐Class Core System (Waters) provided with QDa Detector (Performance), Acquity UPLC PDA eLambda Detector, and Acquity H‐Class with QDa; using C18 1.7 μm (2.1 × 100 mm) column. Samples were monitored under the following conditions: gradient 99% of eluent A for 0.50 min, from 99% to 20% of eluent A in 0.90 min, 1.50 min at 100% of eluent B, 0.10 min to 99% of eluent A (method 2).

### 2.3 General Procedure for Preparation of Target Compounds (5a–n)

A solution of substituted benzaldehyde (**2a‐i**, 1 equiv.), substituted benzylacetoacetate (**3a‐f**, 1 equiv.), urea (**4**, 1.4 equiv.) and *N*‐bromosuccinimide (5 equiv.) in ethanol was heated under reflux for 24 h. Following cooling, the precipitated crude product was collected by filtration and recrystallised from aqueous ethanol to give the product (**5a‐n**) as a white powder.

### 2.4 Benzyl 4‐(2‐Fluoro‐4‐Methoxyphenyl)‐6‐Methyl‐2‐Oxo‐1,2,3,4‐Tetrahydropyrimidine‐5‐Carboxylate (5a)

Synthesised following the general procedure, using 2‐fluoro‐4‐methoxybenzaldehyde (**2a**), urea (**4**) and benzyl acetoacetate (**3a**) to give the desired compound (**5a**) as a white powder (29%). ^1^H‐NMR (CDCl_3_): *δ* 2.43 (s, 3H), 3.80 (s, 3H), 5.03 (d, *J* = 12.5 Hz, 1H), 5.09 (t, *J* = 14.2 Hz, 1H), 5.52 (s, 1H, NH), 5.71 (d, *J* = 2.5 Hz, 1H), 6.60 (dt, *J* = 2.5, 8.9 Hz, 2H), 7.07–7.12 (m, 3H), 7.24–7.30 (m, 3H), 7.93 (s, 1H, NH) ppm. ^13^C‐NMR (CDCl_3_): *δ* 18.71 (CH_3_), 55.60 (CH), 55.69 (CH_3_), 65.75 (CH_2_), 98.56 (ArC), 101.76 (ArCH), 110.03 (ArCH), 121.91 (ArC), 127.79 (ArCH), 127.91 (ArCH), 128.36 (ArCH), 128.73 (ArCH), 136.06 (ArC), 148.21 (ArC), 159.95 (ArC), 160.75 (ArC), 161.91 (ArC), 165.10 (ArC) ppm. ^19^F‐NMR (CDCl_3_): *δ* −117.77 ppm. MS(ESI)^+^: 371.1 [M + H], m.p.: 145°C–147°C. UPLC (method 2): retention time 1.81 min.

### 2.5 3,4‐Dimethoxybenzyl 4‐(2‐Fluoro‐4‐Methoxyphenyl)‐6‐Methyl‐2‐Oxo‐1,2,3,4‐Tetrahydropyrimidine‐5‐Carboxylate (5b)

Synthesised following the general procedure, using 2‐fluoro‐4‐methoxybenzaldehyde (**2a**), urea (**4**) and 3,4‐dimethoxybenzyl acetoacetate (**3b**) to give the desired compound (**5b**) as a white powder (25%). ^1^H‐NMR (CDCl_3_): *δ* 2.42 (s, 3H), 3.79 (s, 3H), 3.80 (s, 3H), 3.88 (s, 3H), 4.96 (d, *J* = 12.1 Hz, 1H), 5.04 (d, *J* = 12.1 Hz, 1H), 5.48 (s, 1H, NH), 5.69 (d, *J* = 2.5 Hz, 1H), 6.63–6.51 (m, 2H), 6.65 (t, *J* = 10.1 Hz, 1H), 6.75 (dt, *J* = 5.0, 8.2 Hz, 2H), 7.06 (dd, *J* = 5.5, 12.2 Hz, 1H), 7.81 (s, 1H, NH) ppm. ^13^C‐NMR (CDCl_3_): *δ* 18.71 (CH_3_), 49.34 (CH_3_), 49.92 (CH), 55.77 (CH_3_), 55.89 (CH_3_), 65.87 (CH_2_), 98.67 (ArC), 109.94 (ArCH), 110.88 (ArCH), 111.45 (ArCH), 120.87 (ArCH), 121.76 (ArCH), 121.87 (ArCH), 128.57 (ArC), 128.71 (ArC), 148.89 (ArCH), 148.92 (ArC), 152.65 (ArC), 159.91 (ArC), 160.61 (ArC), 160.70 (ArC), 161.87 (ArC), 165.17 (ArC) ppm. ^19^F‐NMR (CDCl_3_): *δ* −117.69 ppm. MS(ESI)^+^: 431.2 [M + H^+^], m.p.: 160°C–162°C. HPLC (method 1): retention time 14.45 min.

### 2.6 3,4‐Dimethoxybenzyl‐4‐(4‐Chlorophenyl)‐6‐Methyl‐2‐Oxo‐1,2,3,4‐Tetrahydropyrimidine‐5‐Carboxylate (5c)

Synthesised following the general procedure, using 4‐chlorobenzaldehyde (**2b**), urea (**4**) and 3,4‐dimethoxybenzyl acetoacetate (**3b**) to give the desired compound (**5c**) as a white powder (34%). ^1^H‐NMR (CDCl_3_): *δ* 2.38 (s, 3H), 3.83 (s, 3H), 3.91 (s, 3H), 4.96 (d, *J* = 12.0 Hz, 1H), 5.06 (d, *J* = 12.0 Hz, 1H), 5.39 (d, *J* = 2.2 Hz, 1H), 5.63 (s, 1H, NH), 6.71 (s, 1H), 6.75 (d, *J* = 8.2 Hz, 1H), 6.82 (d, *J* = 8.1 Hz, 1H), 7.19 (d, *J* = 8.4 Hz, 2H), 7.25 (d, *J* = 8.4 Hz, 2H), 7.74 (s, 1H, NH) ppm. ^13^C‐NMR (CDCl_3_): *δ* 18.86 (CH_3_), 55.21 (CH), 55.86 (CH_3_), 55.93 (CH_3_), 66.20 (CH_2_), 100.85 (ArC), 110.98 (ArCH), 111.78 (ArCH), 121.21 (ArCH), 128.08 (ArCH), 128.29 (ArCH), 128.87 (ArC), 133.80 (ArC), 142.05 (ArC), 146.88 (ArC), 148.94 (ArC), 149.12 (ArC), 152.66 (ArC), 165.19 (ArC) ppm. MS(ESI)^+^: 417.1 [M + H]^+^, m.p.: 158°C–160°C. UPLC (method 2): retention time 2.01 min.

### 2.7 Benzo[d][1,3]dioxol‐5‐ylmethyl‐4‐(2‐Fluoro‐4‐Methoxyphenyl)‐6‐Methyl‐2‐Oxo‐1,2,3,4‐Tetrahydropyrimidine‐5‐Carboxylate (5d)

Synthesised following the general procedure, using 2‐fluoro‐4‐methoxybenzaldehyde (**2a**), urea (**4**) and benzo[d][1,3]dioxol‐5‐ylmethylacetoacetate (**3c**) to give the desired compound (**5d**) as a white powder (83%). ^1^H‐NMR (CDCl_3_): *δ* 2.43 (s, 3H), 3.81 (s, 3H), 4.90–4.98 (m, 2H), 5.36 (s, 1H, NH), 5.69 (d, *J* = 2.4 Hz, 1H), 5.96 (dd, *J* = 1.4, 3.1 Hz, 2H), 6.54 (d, *J* = 1.5 Hz, 1H), 6.59–6.68 (m, 3H), 6.71 (d, *J* = 7.9 Hz, 1H), 7.07 (dd, *J* = 6.5, 11.2 Hz, 1H), 7.17 (s, 1H, NH) ppm. ^13^C‐NMR (CDCl_3_): *δ* 18.85 (CH_3_), 49.40 (CH_3_), 55.57 (CH), 65.73 (CH_2_), 98.78 (ArC), 101.74 (ArCH), 101.95 (ArCH), 108.00 (ArCH), 108.67 (ArCH), 110.05 (ArC), 121.83 (ArCH), 128.70 (ArCH), 129.79 (ArC), 147.74 (ArC), 152.11 (ArC), 159.97 (ArC), 160.70 (ArC), 160.91 (ArC), 161.91 (ArC), 165.02 (ArC) ppm. ^19^F‐NMR (CDCl_3_): *δ* −117.82 ppm. MS(ESI)^−^: 415.1 [M−H]^−^, m.p.: 138°C–140°C. HPLC (method 1): retention time 16.2 min.

### 2.8 4‐Methoxy‐3‐Methylbenzyl‐4‐(4‐Chlorophenyl)‐6‐Methyl‐2‐Oxo‐1,2,3,4‐Tetrahydropyrimidine‐5‐Carboxylate (5e)

Synthesised following the general procedure, using 4‐chlorobenzaldehyde (**2b**), urea (**4**) and 4‐methoxy‐3‐methylbenzyl acetoacetate (**3d**) to give the desired compound (**5e**) as a white powder (47%). ^1^H‐NMR (CDCl_3_): *δ* 2.10 (s, 3H), 2.28 (s, 3H), 3.76 (s, 3H), 4.84 (d, *J* = 12.0 Hz, 1H), 4.93 (d, *J* = 12.0 Hz, 1H), 5.28–5.29 (m, 1H), 5.48 (s, 1H, NH), 6.65–6.67 (m, 1H), 6.83 (s, 1H), 6.88–6.90 (m, 1H), 7.08–7.10 (m, 2H), 7.14–7.16 (m, 2H), 7.51 (s, 1H, NH) ppm. ^13^C‐NMR (CDCl_3_): *δ* 16.17 (CH_3_), 18.84 (CH_3_), 55.24 (CH_3_), 55.37 (CH), 66.02 (CH_2_), 100.92 (ArC), 109.63 (ArCH), 127.24 (ArCH), 127.41 (ArC), 128.12 (ArCH), 128.86 (ArCH), 131.03 (ArCH) 133.75 (ArC), 142.06 (ArC), 146.72 (ArC), 152.62 (ArC), 157.76 (C=O), 165.20 (C=O) ppm. MS(ESI)^+^: 401.1 [M + H^+^], 423.1 [M + Na^+^], m.p.: 158°C–160°C. UPLC (method 2): retention time 2.42 min.

### 2.9 Benzyl 4‐(2‐Fluoro‐4‐(Trifluoromethoxy)phenyl)‐6‐Methyl‐2‐Oxo‐1,2,3,4‐Tetrahydropyrimidine‐5‐Carboxylate (5f)

Synthesised following the general procedure, using 2‐fluoro‐4‐trifluoromethoxybenzaldehyde (**2c**), urea (**4**) and benzyl acetoacetate (**3a**) to give the desired compound (**5f**) as a white powder (45%). ^1^H‐NMR (CDCl_3_): *δ* 2.35 (s, 3H), 4.91 (d, *J* = 12 Hz, 1H), 5.04 (d, *J* = 12.5 Hz, 1H), 5.44 (s, 1H), 5.66 (s, 1H, NH), 6.80–6.89 (m, 2H), 6.95–7.05 (m, 2H), 7.05–7.15 (m, 1H), 7.19 (s, 3H), 7.61 (s, 1H, NH) ppm. ^13^C‐NMR (CDCl_3_): *δ* 18.85 (CH_3_), 49.44 (CH), 66.02 (CH_2_), 97.99 (ArC), 109.16 (ArCH), 109.36 (ArCH), 116.74 (ArCH), 127.98 (ArCH), 128.17 (ArCH), 128.43 (ArCH), 129.23 (ArCH), 129.23 (ArCH), 135.76 (ArC), 148.69 (ArC), 152.24 (ArC), 159.20 (ArC), 161.19 (C=O), 164.76 (C=O) ppm. ^19^F‐NMR (CDCl_3_): *δ* −58.03, −115.56 ppm. MS(ESI)^+^: 425 [M + H^+^], m.p.: 195°C–197°C. UPLC (method 2): retention time 2.44 min.

### 2.10 3,4‐Dimethoxybenzyl 4‐(3‐Chlorophenyl)‐6‐Methyl‐2‐Oxo‐1,2,3,4‐Tetrahydropyrimidine‐5‐Carboxylate (5g)

Synthesised following the general procedure, using 3‐chlorobenzaldehyde (**2d**), urea (**4**) and 3,4‐dimethoxybenzyl acetoacetate (**3b**) to give the desired compound (**5g**) as a white powder (52%). ^1^H‐NMR (CDCl_3_): *δ* 2.40 (s, 3H), 3.84 (s, 3H), 3.91 (s, 3H), 5.00 (d, *J* = 12 Hz, 1H), 5.05 (d, *J* = 12 Hz, 1H), 5.36 (s, 1H, NH), 5.39 (s, 1H), 6.73–6.74 (m, 1H), 6.78–6.80 (m, 1H), 6.83–6.85 (m, 1H), 6.89 (s, 1H, NH), 7.14–7.16 (m, 1H), 7.20–7.27 (m, 3H) ppm. ^13^C‐NMR (CDCl_3_): *δ* 18.79 (CH_3_), 55.29 (CH), 55.87 (CH_3_), 55.92 (CH_3_), 66.24 (CH_2_), 100.47 (ArC), 111.03 (ArCH), 111.71 (ArCH), 121.27 (ArCH), 124.83 (ArCH), 126.87 (ArCH), 128.16 (ArCH), 128.26 (ArC), 130.06 (ArCH), 134.53 (ArC), 145.52 (ArC), 147.38 (ArC), 148.93 (ArC), 149.07 (ArC), 153.01 (ArC), 165.19 (C=O) ppm. MS(ESI)^+^: 417.1 [M + H^+^], m.p.: 151°C–153°C. UPLC (method 2): retention time 2.10 min.

### 2.11 3‐Methoxybenzyl 4‐(4‐Chlorophenyl)‐6‐Methyl‐2‐Oxo‐1,2,3,4‐Tetrahydropyrimidine‐5‐Carboxylate (5h)

Synthesised following the general procedure, using 4‐chlorobenzaldehyde (**2b**), urea (**4**) and 3‐methoxybenzyl acetoacetate (**3e**) to give the desired compound **5 h** as a white powder (23%). ^1^H‐NMR (CDCl_3_): *δ* 2.38 (s, 3H), 3.79 (s, 3H), 5.02 (d, *J* = 12 Hz, 1H), 5.09 (d, *J* = 12 Hz, 1H), 5.40 (s, 1H), 5.79 (s, 1H, NH), 6.70 (s, 1H), 6.74–6.76 (m, 1H), 6.85–6.87 (m, 1H), 7.19–7.26 (m, 5H), 8.10 (s, 1H, NH) ppm. ^13^C‐NMR (CDCl_3_): *δ* 18.84 (CH_3_), 55.17 (CH_3_), 55.22 (CH), 65.93 (CH_2_), 100.66 (ArC), 113.54 (ArCH), 113.74 (ArCH), 120.33 (ArCH), 128.07 (ArCH), 128.92 (ArCH), 129.55 (ArCH), 133.83 (ArC), 137.34 (ArC), 142.01 (ArC), 147.23 (ArC), 152.92 (ArC), 159.69 (C=O), 165.10 (C=O) ppm. MS(ESI)^+^: 387.1 [M + H^+^], m.p.: 120°C–122°C. UPLC (method 2): retention time 2.28 min.

### 2.12 4‐Methoxybenzyl 4‐(4‐Chlorophenyl)‐6‐Methyl‐2‐Oxo‐1,2,3,4‐Tetrahydropyrimidine‐5‐Carboxylate (5i)

Synthesised following the general procedure, using 4‐chlorobenzaldehyde (**2b**), urea (**4**) and 4‐methoxybenzyl acetoacetate (**3f**) to give the desired compound **5i** as a white powder (48%). ^1^H‐NMR (CDCl_3_): *δ* 2.37 (s, 3H), 3.84 (s, 3H), 4.97 (d, *J* = 12 Hz, 1H), 5.05 (d, *J* = 12 Hz, 1H), 5.38 (s, 1H), 5.58 (s, 1H, NH), 6.85–6.86 (m, 2H), 7.10–7.12 (m, 2H), 7.17–7.19 (m, 2H), 7.24–7.26 (m, 2H), 7.62 (s, 1H, NH) ppm. ^13^C‐NMR (CDCl_3_): *δ* 18.88 (CH_3_), 55.26 (CH_3_), 55.30 (CH), 65.88 (CH_2_), 100.91 (ArC), 113.84 (ArCH), 127.89 (ArC), 128.09 (ArCH), 128.88 (ArCH), 130.00 (ArCH), 133.77 (ArC), 142.03 (ArC), 146.74 (ArC), 152.53 (ArC), 159.60 (C=O), 165.18 (C=O) ppm. ^19^F‐NMR (CDCl_3_): *δ* −117.79 ppm. MS(ESI)^+^: 387.1 [M + H^+^], m.p.: 161°C–163°C. UPLC (method 2): retention time 2.26 min.

### 2.13 3,4‐Dimethoxybenzyl 4‐(2‐Chlorophenyl)‐6‐Methyl‐2‐Oxo‐1,2,3,4‐Tetrahydropyrimidine‐5‐Carboxylate (5j)

Synthesised following the general procedure, using 2‐chlorobenzaldehyde (**2e**), urea (**4**) and 3,4‐dimethoxybenzyl acetoacetate (**3b**) to give the desired compound **5j** as a white powder (35%). ^1^H‐NMR (CDCl_3_): *δ* 2.49 (s, 3H), 3.79 (s, 3H), 3.88 (s, 3H), 4.97 (d, *J* = 12.5 Hz, 1H), 5.00 (d, *J* = 12 Hz, 1H), 5.59 (s, 1H, NH), 5.92 (s, 1H), 6.61 (s, 1H), 6.65–6.67 (m, 1H), 6.73–6.75 (m, 1H), 7.17 (s, 1H, NH), 7.22–7.24 (m, 3H), 7.36–7.38 (m, 1H) ppm. ^13^C‐NMR (CDCl_3_): *δ* 18.49 (CH_3_), 52.17 (CH), 55.78 (CH_3_), 55.90 (CH), 65.85 (CH_2_), 98.49 (ArC), 110.85 (ArCH), 111.23 (ArCH), 120.68 (ArCH), 127.56 (ArCH), 127.93 (ArCH), 128.49 (ArC), 129.34 (ArCH), 129.85 (ArCH), 132.65 (ArC), 139.31 (ArC), 148.81 (ArC), 148.84 (ArC), 149.04 (ArC), 152.85 (ArC), 165.06 (C=O) ppm. MS(ESI)^+^: 417.1 [M + H^+^], m.p.: 174°C–176°C. UPLC (method 2): retention time 2.14 min.

### 2.14 Benzo[d] [1,3] Dioxol‐5‐ylmethyl 4‐(4‐Methoxyphenyl)‐6‐Methyl‐2‐Oxo‐1,2,3,4‐Tetrahydropyrimidine‐5‐Carboxylate (5k)

Synthesised following the general procedure, using 4‐methoxybenzaldehyde (**2f**), urea (**4**) and compound (**3c**) to give the desired compound **5k** as a white powder (70%). ^1^H‐NMR (CDCl_3_): *δ* 2.36 (s, 3H), 3.81 (s, 3H), 4.91 (d, *J* = 12 Hz, 1H), 5.01 (d, *J* = 12 Hz, 1H), 5.33–5.37 (m, 1H), 5.73 (s, 1H, NH), 5.94–5.98 (m, 2H), 6.59–6.61 (m, 1H), 6.65–6.69 (m, 1H), 6.74 (d, *J* = 8 Hz, 1H), 6.82 (d, *J* = 9 Hz, 2H), 7.18 (d, *J* = 8.5 Hz, 2H), 8.21 (s, 1H, NH) ppm. ^13^C‐NMR (CDCl_3_): *δ* 18.71 (CH_3_), 55.21 (CH_3_), 55.26 (CH), 65.81 (CH_2_), 101.11 (CH_2_), 108.06 (ArCH), 108.88 (ArCH), 114.04 (ArCH), 121.99 (ArCH), 127.88 (ArCH), 129.81 (ArC), 135.96 (ArC), 146.73 (ArC), 147.43 (ArC), 47.67 (ArC), 153.10 (ArC), 159.31 (C=O), 165.40 (C=O) ppm. MS(ESI)^+^: 397.2 [M + H^+^], m.p.: 141°C–143°C. UPLC (method 2): retention time 2.07 min.

### 2.15 Benzyl 4‐(4‐Fluorophenyl)‐6‐Methyl‐2‐Oxo‐1,2,3,4‐Tetrahydropyrimidine‐5‐Carboxylate (5l)

Synthesised following the general procedure, using 4‐fluorobenzaldehyde (**2g**), urea (**4**) and benzyl acetoacetate (**3a**) to give the desired compound (**5l**) as a white powder (22%). ^1^H‐NMR (CDCl_3_): *δ* 2.38 (s, 3H), 5.03 (d, *J* = 12.5 Hz, 1H), 5.12 (d, *J* = 12.5 Hz, 1H), 5.41 (s, 1H), 5.74 (s, 1H, NH), 6.96 (t, *J* = 17 Hz, 2H), 7.13–7.18 (m, 2H), 7.29 (s, 1H), 7.30–7.35 (m, 3H), 7.39 (s, 1H), 7.94 (s, 1H, NH) ppm. ^13^C‐NMR (CDCl_3_): *δ* 18.86 (CH_3_), 55.16 (CH), 66.06 (CH_2_), 115.51 (ArCH), 115.69 (ArCH), 128.14 (ArCH), 128.18 (ArCH), 128.49 (ArCH), 135.84 (ArC), 139.46 (ArC), 146.97 (ArC), 152.81 (ArC), 161.40 (ArC), 163.37 (C=O), 165.21 (C=O) ppm. ^19^F‐NMR (CDCl_3_): *δ* −144.13 ppm. MS(ESI)^+^: 341.1 [M + H^+^], m.p.: 141°C–143°C. UPLC (method 2): retention time 2.18 min.

### 2.16 Benzyl 4‐(2‐Fluorophenyl)‐6‐Methyl‐2‐Oxo‐1,2,3,4‐Tetrahydropyrimidine‐5‐Carboxylate (5m)

Synthesised following the general procedure, using 2‐fluorobenzaldehyde (**2h**), urea (**4**) and compound benzyl acetoacetate (**3a**) to give the desired compound (**5 m**) as a white powder (39%). ^1^H‐NMR (CDCl_3_): *δ* 2.44 (s, 3H), 5.03 (d, *J* = 12.5 Hz, 1H), 5.09 (d, *J* = 12.5 Hz, 1H), 5.65 (s, 1H), 5.79 (s, 1H, NH), 7.00–7.12 (m, 4H), 7.22–7.33 (m, 5H), 8.21 (s, 1H, NH) ppm. ^13^C‐NMR (CDCl_3_): *δ* 18.67 (CH_3_), 49.58 (CH), 65.79 (CH_2_), 115.85 (ArCH), 124.48 (ArCH), 128.22 (ArCH), 128.38 (ArCH), 129.54 (ArCH), 129.65 (ArCH), 129.71 (ArCH), 129.78 (ArC), 136.00 (ArC), 148.75 (ArC), 152.90 (ArC), 159.39 (ArC), 161.35 (C=O), 165.05 (C=O) ppm. ^19^F‐NMR (CDCl_3_): *δ* −112.25 ppm. MS(ESI)^+^: 341.1 [M + H^+^], m.p.: 155°C–157°C. UPLC (method 2): retention time 2.17 min.

### 2.17 Benzyl 4‐(2‐Fluoro‐4‐Methylphenyl)‐6‐Methyl‐2‐Oxo‐1,2,3,4‐Tetrahydropyrimidine‐5‐Carboxylate (5n)

Synthesised following the general procedure, using 2‐fluoro‐4‐methylbenzaldehyde (**2i**), urea (**4**) and benzyl acetoacetate (**3a**) to give the desired compound (**5n**) as a white powder (82%). ^1^H‐NMR (CDCl_3_): *δ* 2.35 (s, 3H), 2.43 (s, 3H), 5.03 (d, *J* = 12.5 Hz, 1H), 5.09 (d, *J* = 12.5 Hz, 1H), 5.60 (s, 1H, NH), 5.70–5.79 (m, 1H), 6.80–6.90 (m, 2H), 7.00–7.15 (m, 3H), 7.25–7.33 (m, 3H), 8.18 (s, 1H, NH) ppm. ^13^C‐NMR (CDCl_3_): *δ* 18.68 (CH_3_), 21.06 (CH_3_), 49.40 (CH), 65.73 (CH_2_), 98.33 (ArC), 116.18 (ArCH), 116.36 (ArCH), 125.06 (ArCH), 127.77 (ArCH), 127.90 (ArCH), 128.36 (ArCH), 136.09 (ArC), 140.29 (ArC), 148.52 (ArC), 152.89 (ArC), 159.23 (ArC), 161.19 (C=O), 165.12 (C=O) ppm. ^19^F‐NMR (CDCl_3_): *δ* −120.92 ppm. MS(ESI)^+^: 355.1 [M + H^+^], m.p.: 184°C–186°C. UPLC (method 2): retention time 2.26 min.

### 2.18 General Cell Culture

Human Embryonic Kidney cells (HEK‐293) and human breast cancer cells (MCF‐7, MDA‐MB‐231 and HCC1954) were cultured in complete growth media: RPMI‐1600 1X (Gibco); 10% v/v Fetal Bovine Serum (FBS, Sigma Dorset UK) and l‐glutamine (2 mM, Invitrogen). SW‐480‐WT cells were maintained in selective media made up of Dulbecco's Modified Eagle Medium (Gibco), enriched with 1000 μg/μL of Geneticin Selective Antibiotic (G418 Sulfate; Gibco, Thermo Fisher). Cells were incubated at a 5% CO_2_ atmosphere at 37°C.

### 2.19 CellTiter‐Blue Assay

Cells were cultured in 96‐well plates in 90 μL at a seeding density of 1 × 10^6^ cells per well. Compound samples were prepared at different concentrations (1 mM, 100 μM, 10 μM, 1 μM, 100 nM, 10 nM) and 10 μL of each concentration was seeded in each well. Two different negative controls were used: DMSO 0.01% and 0.001%. Paclitaxel (30% w/v) was used as a positive control. Cells were treated for 24 h after which 20 μL of CellTitre‐Blue reagent (Promega, Southampton, UK) was added and incubated for 2 h at 37°C in 5% CO_2_. Fluorescence was measured at 560/590 nm on a Fluorostar Optima plate reader (BMG Labtech, Bucks, UK). Assay end points were normalised to 0% (DMSO only) and fitted to a semilog plot using at least *n* = 3 technical replicates (*n* = 6 for DMSO control and paclitaxel control) and *N* = 3 independent replicates. The curve fit was performed using the GraphPad Prism software and the standard Hill equation.

### 2.20 Colony Forming Assay

Cells were seeded in a 12‐well plate at 400 cells per well. One hour after the seeding of the cells, compounds (1 mM, 100 μM, 10 μM, 1 μM, 100 nM, 10 nM) or vehicle were added into the wells in triplicate (DMSO vehicle 0.1% and 1%). After 7 days, colonies were stained with 350 μL of crystal violet solution, incubated for 30 min at room temperature, then wells were washed in water and dried. At least the best 3 independent experiments out of 5 independent repeats were used to perform the statistical analysis of the results. Colonies were counted by GelCount Colony Counter (Oxford Optronix Ltd.) using the GelCount 1.1.8.0 software. Assay end points were normalised to 0% (DMSO only) and fitted to a semi‐log plot using at least *n* = 3 technical replicates (*n* = 6 for DMSO control and *N* = 3 independent replicates). The curve fit was performed using GraphPad Prism.

### 2.21 Transcreener ADP Fluorescence Polarisation (FP) Assay

Transcreener ADP (cat. no. 3004‐1K) and ADP2 (cat. no. 3010‐1K) FP assay kits were obtained from Bellbrook Laboratories. Recombinant human Hsp90 alpha protein (active), human full‐length protein, expressed in *Escherichia coli* with > 90% purity and C‐terminus His tag, was purchased from Abcam (ab80369). All FP assays were carried out in black, 384‐well, low‐volume, polystyrene nonbinding microplates from Corning Life Sciences. All basic buffer components were obtained from Sigma‐Aldrich Chemical Co. 17‐AAG (Figure 1) was obtained from Abcam. Using the Transcreener ADP FP kit, the conditions for the recombinant human Hsp90 ATPase reaction were 50 mM HEPES (pH 7.4), 20 mM KCl, 2 mM EGTA, 4 mM MgCl_2_, and 0.01% Brij‐35 in a 10‐μL total assay volume. The final assay concentration of ATP was 100 μM for Hsp90. Due to the relatively low level of ATPase activity compared with other enzymes, the plate was incubated at 37°C and run over 1, 2 and 3 h time points. The reaction was stopped, and the ADP was detected by adding 10 μL of 50 mM HEPES (pH 7.4), 400 mM NaCl, 20 mM EDTA, 0.01% Brij35, 75 μg/mL anti‐ADP monoclonal antibody, and 4 nM ADP far red tracer. The plate was sealed, centrifuged at 600 *g* for 1 min, and left at room temperature for 1 h. FP readings for the Hsp90 ATPase Transcreener assays were performed on a Clariostar plate reader (PerkinElmer Life Sciences). A blank mP signal composed of protein, antibody, labelled ADP, but no ATP substrate gave similar mP values to those seen in the no‐protein controls. The upper limit of the assay window was defined by a no‐protein control, and the mP in the presence of heat shock protein was subtracted from this control to generate a change in polarisation (ΔmP), which was plotted on the activity graphs. Therefore, the data take into account the spontaneous hydrolysis of ATP. Each value is the mean of triplicate determinations with standard deviations (SD) shown. IC_50_ values were calculated using GraphPad Prism software with the nonlinear regression curve fitted to the log (inhibitor concentration) against the raw mP values on the linear portion of the standard curve.

## 3 Results

### 3.1 Computational Docking

Computational docking experiments were carried out to assess the interaction of virtual hit compound **1** (S‐enantiomer) within the Hsp90 ATP binding site (PDB code: 1YET). Compound **1** was able to mimic some of the important interactions of geldamycin within its binding pocket (Stebbins et al. 1997). As shown in Figure 2, compound **1** is stabilised by three interactions with three different key amino acid residues in the ATP binding pocket: lysine 112, glycine 135 (both making interactions with the dihydropyrimidinone core) and asparagine 64 (interaction with the benzyloxy side chain).

### 3.2 Chemical Synthesis

**SCHEME 1:**
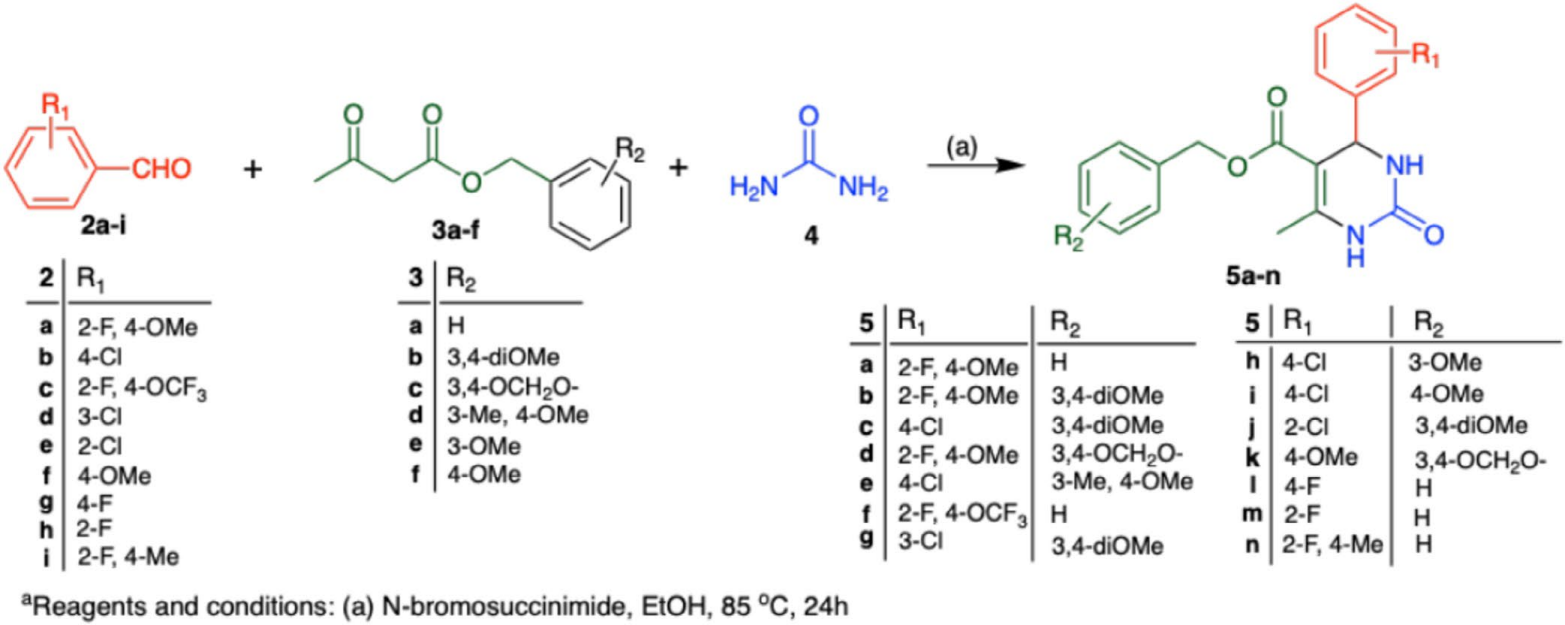
Synthesis of 4‐aryl‐3,4‐dihydropyrimidin‐2‐one‐5‐carboxylates.^a^

Based on the discovery of the virtual hit compound **1**, we designed a structure–activity relationship (SAR) study to optimise activity around the 4‐aryl‐3,4‐dihydropyrimid‐2‐one‐5‐carboxylate scaffold, based on the multicomponent Biginelli reaction to efficiently construct the core structure (Scheme 1) (Kappe and Stadler 2004). Our SAR approach was designed to test the effect of different electron‐withdrawing and electron‐donating substituents on the 4‐phenyl and benzyl ester rings. Biological readouts for the SAR study included inhibition of colony formation within human cancer cell lines, selective inhibition of cell viability in cancer cell lines, inhibition of Hsp90 activity, and in vitro assessment of pharmacokinetic parameters (solubility and metabolic stability).

The multicomponent Biginelli reaction is shown in Scheme 1, bringing together substituted aldehyde (**2a–i**), β‐ketoester (**3a–f**) and urea (**4**) in the presence of N‐bromosuccinimide (NBS) catalyst in ethanol at 85°C (22%–83% isolated yields) (Hazarkhani and Karimi 2004). The Biginelli reaction proceeds through the mechanism that begins with the nucleophilic addition of an aldehyde and urea, forming an N‐(1‐hydroxybenzyl)urea intermediate. This intermediate subsequently undergoes dehydration, generating the N‐acyliminium ion, which then reacts with a β‐keto ester. The reaction culminates in a cyclisation step, producing the Biginelli product, dihydropyrimidinone. Spectroscopic evidence supports the formation of these products, with key proton NMR signals providing identification. Notably, the α‐hydrogen appears as a singlet in the 5.33–5.92 ppm range, integrating to one proton; additionally, the 6‐methyl group is observed as a singlet integrating to three protons with a chemical shift between 2.28 and 2.49 ppm. Whilst the majority of starting materials were commercially available, it was necessary to synthesise certain β‐keto esters (**3a–f**) using previously established procedures (see Supporting Information).

### 3.3 Cell Cytotoxicity and Inhibition of Colony Formation

In view of previous reports on Hsp90 inhibitors and their effects on cancer cell viability (Eachkoti et al. 2014) and clonogenicity (Kinzel et al. 2016), new compounds were tested for inhibition of cell viability within both non‐tumourigenic (HEK‐293) and breast cancer cell lines (MDA‐MB‐231) at a fixed concentration of 10 μM. The well‐established CellTiter‐Blue endpoint assay was used to assess cell viability. New compounds were also assessed in a colony forming assay (MCF‐7 breast cancer cell line), used as an efficacy screening to provide an indication of the inhibition of clonogenicity for these small molecules (Table 1).

**TABLE 1:** Cell cytotoxicity and colonogenicity for selected compounds (**5a–n**).

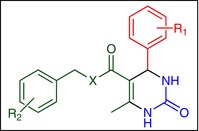					
Cpd	R_1_	R_2_	% Viability (HEK‐293, 10 μM)^a^	% Viability (MDA‐MB‐231, 10 μM)^a^	% Clonogenicity (MCF‐7, 10 μM)^b^
**5a**	2‐F, 4‐OMe	H	90.62 ± 3.35	91.33 ± 0.64	71.87 ± 4.77
**5b**	2‐F, 4‐OMe	3,4‐diOMe	99.92 ± 1.50	89.67 ± 1.37	71.39 ± 2.94
**5c**	4‐Cl	3,4‐diOMe	99.41 ± 3.16	73.55 ± 3.50	64.29 ± 3.69
**5d**	2‐F, 4‐OMe	3,4‐OCH_2_O	99.92 ± 4.53	86.29 ± 3.80	49.75 ± 4.35
**5e**	4‐Cl	3‐Me‐4‐OMe	82.07 ± 1.28	79.43 ± 8.8	59.96 ± 1.95
**5f**	2‐F, 4‐OCF_3_	H	95.11 ± 3.82	85.73 ± 2.65	72.41 ± 3.21
**5g**	3‐Cl	3,4‐diOMe	96.71 ± 3.77	72.16 ± 7.20	50.04 ± 2.3
**5h**	4‐Cl	3‐OMe	81.00 ± 2.36	88.05 ± 3.10	42.34 ± 4.80
**5i**	4‐Cl	4‐OMe	94.32 ± 2.95	83.26 ± 0.69	8.39 ± 2.84
**5j**	2‐Cl	3,4‐diOMe	92.96 ± 2.59	76.50 ± 4.28	58.18 ± 4.42
**5k**	4‐OMe	3,4‐OCH_2_O	98.62 ± 1.31	98.66 ± 2.60	99.85 ± 3.27
**5l**	4‐F	H	98.02 ± 1.89	100.03 ± 2.96	82.62 ± 2.93
**5m**	2‐F	H	97.74 ± 3.27	95.17 ± 3.72	99.95 ± 2.40
**5n**	2‐F, 4‐Me	H	98.71 ± 2.49	97.35 ± 1.30	90.54 ± 0.81

^a^ Cell viability (CellTiter‐Blue) represents the percentage relative fluorescence unit (24 h incubation time, 10 μM test compound; value standardised to DMSO control).

^b^ Colony forming inhibition represents the percentage of survival colonies after treatment with target compound at various concentrations (7‐day incubation time, value standardised to DMSO control). Percentage values for the colony formation assay were generated using GraphPad prism software and are represented as the mean of three replicates with errors reported as the standard deviation (*N* = 3, *n* = 3, error reported as standard deviation).

Within the non‐tumourigenic HEK‐293 kidney epithelial cell line, 24 h treatment with test compounds at 10 μM resulted in only minor cytotoxic effects, with > 90% relative to the negative control. The exceptions in HEK‐293 cells where cell viability was lower were the dihydropyrimidinones **5e** (R_1_ = 4‐Cl, R_2_ = 3‐Me, 4‐OMe; 82% cell viability) and **5h** (R_1_ = 4‐Cl, R_2_ = 3‐OMe; 81% cell viability). Considering the breast cancer cell line MDA‐MB‐231 (expressing high endogenous levels of Hsp90), a range of compounds exhibited significant inhibition of cell viability at 10 μM treatment concentration. Most notably, treatment with compounds **5c** (R_1_ = 4‐Cl, R_2_ = 3,4‐diOMe), **5e** (R_1_ = 4‐Cl, R_2_ = 3‐Me, 4‐OMe), **5g** (R_1_ = 3‐Cl, R_2_ = 3,4‐diOMe) and **5j** (R_1_ = 2‐Cl, R_2_ = 3,4‐diOMe) gave a decrease in viability (< 80%). The colony inhibition assay (10 μM treatment over 7 days) using the MCF‐7 breast cancer cell line was particularly instructive as a measure of Hsp90's on‐target effects on clonogenicity, with the most notable reduction in colony formation (< 50% remaining) for test compounds **5d** (R_1_ = 2‐F, 4‐OMe, R_2_ = 3,4‐OCH_2_O‐; 49.75%) and **5i** (R_1_ = 4‐Cl, R_2_ = 4‐OMe; 8.39%). Compound **5h** (R_1_ = 4‐Cl, R_2_ = 3‐OMe) exhibited a decrease in the percentage of colonies in the MCF‐7 cell line, but this compound was also found to be more growth inhibitory than the others in the HEK‐293 cell line. This suggested that the effect of compound **5h** might be due to non‐specific reduction in cell viability rather than an on‐target effect.

Table 2 compares the CellTiter‐Blue assay GI_50_ values (HEK‐293 and MDA‐MB‐231 human cell lines, 24 h incubation time) and colony formation data IC_50_ values (MCF‐7 breast cancer cells) with the Hsp90 ATPase activity IC_50_ values for each of the compounds.

**TABLE 2:** IC_50_ calculation for test compounds (**5a–n**) within in vitro cell‐based (HEK‐293 and MDA‐MB‐231) and Hsp90 ATPase inhibitory assays.

Compounds	CellTiter‐Blue assay GI_50_ (μM)^a^		Colony forming assay IC_50_ (μM)^a^	ATPase assay on rh‐Hsp90 IC_50_ (μM)^a^
	HEK‐293	MDA‐MB‐231	MCF‐7	Recombinant human HSP90
**5a**	71.55	86.18	5.03	5.36
**5b**	44.92	72.09	0.68	13.09
**5c**	12.60	22.71	2.46	4.28
**5d**	92.39	79.30	0.29	3.25
**5e**	86.30	12.59	0.91	6.40
**5f**	16.41	98.93	2.29	13.67
**5g**	39.05	19.28	0.83	32.63
**5h**	12.27	23.73	9.081	6.47
**5i**	98.45	39.98	0.56	9.59
**5j**	58.02	65.66	3.49	32.51
**5k**	62.98	77.24	2.46	20.23
**5l**	52.92	86.63	0.89	22.92
**5m**	20.45	84.89	15.67	16.62
**5n**	39.59	86.65	7.17	74.55

^a^ IC_50_ values were generated using GraphPad prism software and are represented as the mean of three replicates (*N* = 3, *n* = 3).

**FIGURE 3:**
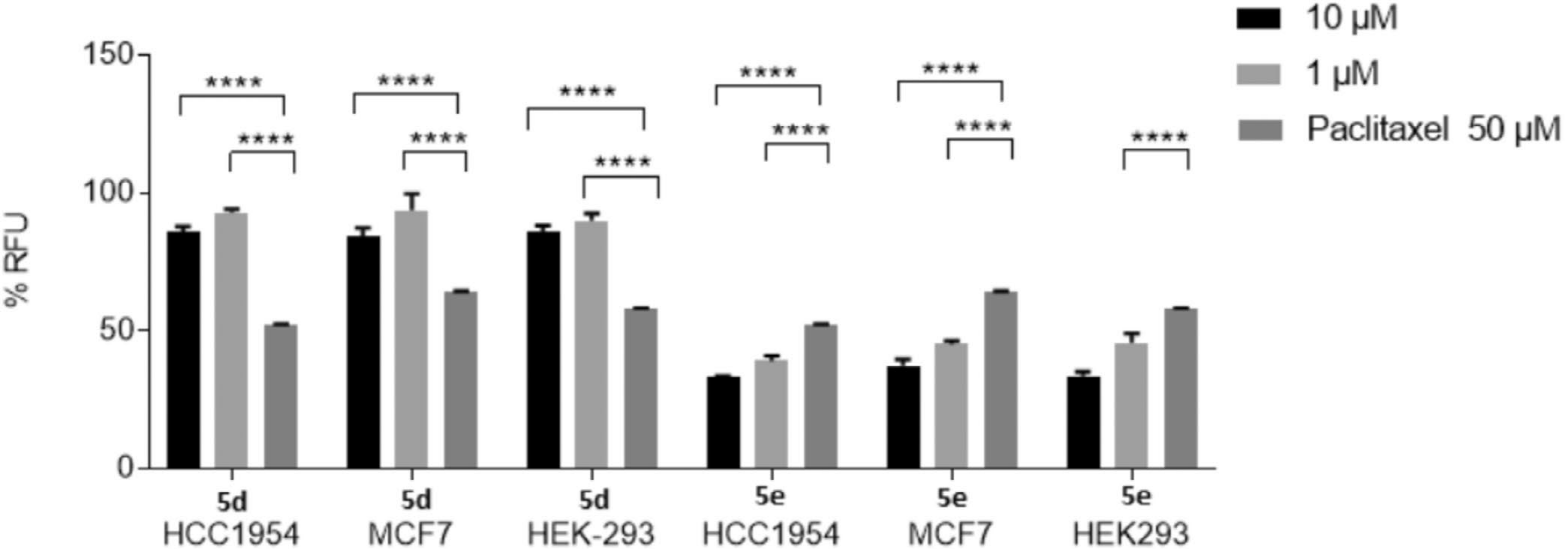
Growth inhibition assay (CellTitre‐Blue endpoint) in human breast cancer cell lines HCC1954 and MCF‐7, and non‐tumorigenic HEK‐293 cells for hit compounds **5d** and **5e** (72 h incubation). Growth inhibition is represented by the percentage of relative fluorescence units standardised to DMSO control. Values are the mean of three independent experiments carried out in triplicate (*N* = 3, *n* = 3, ± standard deviation).

Compound **5d** (R_1_ = 2‐F, 4‐OMe, R_2_ = 3,4‐OCH_2_O) was found to potently inhibit colony formation, coupled with potent (low micromolar IC_50_) Hsp90 inhibition, without exhibiting significant effects on cell viability (Table 2). Compound **5a** (R_1_ = 2F, 4‐OMe, R_2_ = H) largely retained cell viability in HEK‐293 and MDA‐MB‐231 cell lines; plus, single‐digit micromolar potency in the MCF‐7 colony‐forming assay, and the ATPase Hsp90 assay. Compound **5i** (R_1_ = 4‐Cl, R_2_ = 4‐OMe) similarly showed potent activity in the MCF‐7 colony‐forming and Hsp90 inhibition assays but had more potent effects on cell viability in MDA‐MB‐231 cells (IC_50_ = 40 μM). Replacing the methoxy group of compound **5d** with the electron‐withdrawing OCF_3_ group in the R_1_ position (compound **5f**) led to a less active and more cytotoxic (HEK‐293 cells) compound. Analogues **5b** and **5l** were found to inhibit colony formation at sub/low‐micromolar IC_50_ concentrations with moderately potent Hsp90 ATPase inhibitory activity but were also found to exhibit weak cytotoxic effects on the non‐tumorigenic HEK‐293 cell line and were not considered further. Compound **5n** (R_1_ = 2‐F, 4‐Me, R_2_ = H) was found to be the least active of the series for Hsp90 ATPase activity. Despite potent activity in the colony formation and Hsp90 ATPase assays, compound **5c** (R_1_ = 4‐Cl, R_2_ = 3,4‐diOMe) had an undesirable cytotoxicity profile in both tumorigenic and non‐tumorigenic cell lines and was discarded due to likely off‐target effects. Introduction of a 3‐methyl‐4‐methoxyphenyl function in the R_2_ position (**5e**) showed selective growth inhibitory activity in MDA‐MB‐231 cells, coupled with potent colony formation and Hsp90 ATPase inhibitory activity, and compound **5e** was considered further. Finally, the analogue containing a 3‐chlorophenyl group in R_1_ (**5g**) showed potent inhibition of colony formation, but non‐selective inhibition of cell viability in both the tumorigenic (MDA‐MB‐231) and non‐tumorigenic (HEK‐293) cell lines, and a relative lack of activity in the ATPase Hsp90 assay suggesting again off‐target activities of the compound. Compounds **5d** and **5e** with the most interesting overall profile (the most potent inhibition of colony formation and Hsp90 activity from the series, coupled with retention of cell viability in HEK‐293 cells) were further evaluated for their effects on cell viability in the additional breast cancer cell lines HCC 1954 and MCF‐7. In this instance, cells were treated for 3 days before CellTiter‐Blue analysis to determine longer term effects of compounds on cell viability, compared with paclitaxel as a positive control (Figure 3).

With a longer incubation time, the non‐specific growth inhibitory effect of compound **5e** became more pronounced and was found to be comparable to the cytotoxic drug control paclitaxel. For these reasons, subsequent experiments focused on the Hsp90‐inhibitory active molecule **5d** devoid of significant effects on cell viability.

Compound **5d** was screened for the ability to inhibit colony formation in additional human cancer cell lines (Table 3). Although **5d** did not significantly inhibit colony formation in the breast cancer cell lines SKBR3 (ER^−^, PR^−^, HER2^+^) and BT474 (ER^+^, PR^+^, HER2^+^), potent effects on surviving colonies were noted in HCC1954 and MDA‐MB‐231 cell lines at 10 μM. Sub‐micromolar IC_50_ values in HCC1954 and MDA‐MB‐231 cells were derived by testing over a six‐fold log concentration range. To further extend the profile of activity, the effect of **5d** on the colorectal adenocarcinoma cell line SW‐480 was studied, with similarly potent effects on colony viability (Table 3).

**TABLE 3:** Inhibition of colony formation for compound **5d** in four breast cancer cell lines (SKBR3, BT474, MDA‐MB‐231, HCC1954) and the colorectal adenocarcinoma cell line SW‐480.

Cell line	Percentage of surviving colonies^a^	IC_50_ (μM)^b^
SKBR3	94.4% ± 2.1	N.A.
BT474	80.4% ± 8.0	N.A.
SW‐480	57.9% ± 2.0	1.21
MDA‐MB‐231	11.9% ± 2.9	0.62
HCC1954	8.8% ± 4.0	0.56

^a^ Percentage of surviving colonies is represented as the mean of three replicates with errors reported as the standard deviation (*N* = 3, *n* = 3) after 7 days treatment at 10 μM.

^b^ IC_50_ values were generated using GraphPad Prism software and are represented as the mean of three replicates (*N* = 3, *n* = 3). N.A. = IC_50_ not achieved over the dosing range.

All the selected analogues were calculated to be compliant with the Lipinski rule‐of‐five guidelines for oral bioavailability. Furthermore, in vitro ADME (absorption, distribution, metabolism, excretion) properties were assessed as an outsourced service (Cyprotex Discovery Ltd., UK; part of the EvoTec Group); profiling membrane permeability (Caco‐2 cell model), hERG channel cardiotoxicity, human microsomal stability and turbidometric solubility (Table 4). Whilst the Caco‐2 permeability efflux ratio (> 2) was suggestive of active efflux, other parameters gave further confidence in compound **5d** (or the structurally related active analogue **5e**) as a lead compound for further development. In particular, the initial hERG assay indicated low potential cardiotoxicity (IC_50_ = > 25 μM), and metabolic half‐life in human microsomes and solubility gave moderate stability and solubility values for further SAR optimization and development.

**TABLE 4:** Early in vitro profiling of ADME properties for hit compounds **5d** and **5e**.

Compound	Caco‐2 permeability	hERG channel	Metabolic stability (human microsomes)			Solubility—estimated precipitation range (μM)		
	Efflux ratio	IC_50_ (μM)	CL_int_ (μL/min/mg protein)	SE CL_int_	Half‐life (min)	Lower‐bound	Upper‐bound	Calc. mid‐range
**5d**	2.26	> 25	34.6	4.05	40.1	1	6.5	3.75
**5e**	2.74	> 25	108	4.86	12.8	30	100	65

In conclusion, the virtual hit compound **1** was found to mimic the interactions of geldanamycin within the Hsp90 ATP binding pocket, stabilised by three key residues: Lys‐112, Gly‐135 and Asp‐54. SAR optimization around the 4‐aryl‐3,4‐dihydropyrimid‐2‐one‐5‐carboxylate scaffold employed the Biginelli reaction for efficient core construction to build a small library of compounds. We assessed the in vitro anticancer potential of analogues via cell viability assays, inhibition of colony formation, Hsp90 inhibition and pharmacokinetic assessment (solubility and stability). Compound **5d** emerged as the most potent compound, exhibiting minimal effects on cell viability and effective Hsp90 inhibition. Compound **5e** showed promising results, similar to control drug paclitaxel in terms of cytotoxicity but showed non‐specific growth inhibition in HEK‐293 cells over prolonged incubation. Further evaluation of compound **5d** in human cancer cell lines demonstrated its potent inhibition of colony formation in breast cancer cell lines (MDA‐MB‐231, HCC1954) and SW‐480 (colorectal cancer), confirming its anti‐cancer potential. Compound **5d** emerged as the most promising compound for further development due to its inhibition of colony formation, low growth inhibitory effects and effective Hsp90 inhibition. In addition, compound **5d** exhibited low potential for cardiotoxicity and moderate stability and solubility, suggesting promise for further development. Further optimization of SAR and pharmacokinetic properties is needed for the advancement of hit compound **5d** into a candidate for further development.

## Funding

This study was supported by Tiziana Life Sciences and Myristica Trust.

## Conflicts of Interest

The authors declare no conflicts of interest.

## Supporting information


**Data S1:** cbdd70221‐sup‐0001‐Supinfo1.docx.

## Data Availability

The data that supports the findings of this study are available in Supporting Information of this article.
