# Supplementary material for: New Anticancer 4‐Aryldihydropyrimidinone‐5‐Carboxylates Targeting Hsp90
Source: Chem Biol Drug Des. 2025 Dec 15;106(6):e70221. doi: 10.1111/cbdd.70221 (PMC12704579; doi:10.1111/cbdd.70221)

**Supporting information**

**New Anticancer 4-Aryldihydropyrimidinone-5-carboxylatesTargeting Hsp90**

Cinzia Bordoni,^1,2^ Tia Z. Elstow,^1^ Andrea Brancale,^1,3^ Richard W.E. Clarkson,^2^ Andrew D. Westwell^*1^

^1^School of Pharmacy and Pharmaceutical Sciences, Cardiff University, Redwood Building, King Edward VII Avenue, Cardiff, CF10 3NB, Wales, U.K.

^2^European Cancer Stem Cell Research Institute, Hadyn Ellis Building, Maindy Road, Cardiff, CF24 4HQ, Wales, U.K.

^3^Department of Organic Chemistry, University of Chemistry and Technology, 16628 Prague, Czech Republic.

**Contents:**

1. Preparative methods and analytical/spectroscopic data for non-commercially available beta-keto esters.
2. Purity (HPLC) of active final compounds.
3. NMR spectra (^1^H and ^13^C) of active final compounds.
4. **Preparative methods and analytical/spectroscopic data for non-commercially available beta-keto esters.**

The reaction scheme for the preparation of benzyl 3-oxobutanoates (**3a-f**) is reported below:


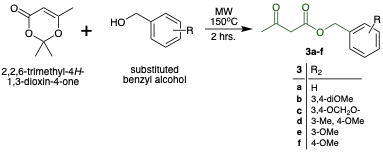


**General Procedure:** A homogenous solution of 2,2,6-trimethyl-4*H*-1,3-dioxin-4-one (1.6 equiv.) and substituted benzyl alcohol (1.0 equiv.) was heated to 150 ^o^C for 2 hours in a CEM Discover Microwave synthesiser. Following cooling, the residue was purified using flash column chromatography (ethyl acetate / hexane eluents) to afford the desired substituted beta-keto ester as an oil.

**Benzyl 3-oxobutanoate (3a):** commercially available (Merck), CAS#: 38432-58-5.

**3,4-Dimethoxybenzyl 3-oxobutanoate (3b).** Colourless oil (80% yield). **^1^H NMR (CDCl_3_):** δ 2.13 (s, 3H), 3.39 (s, 2H), 3.76 (s, 3H), 3.78 (s, 3H), 5.01 (s, 2H), 6.75 (s, 1H), 6.82 (s, 2H) ppm. **^13^C NMR (CDCl_3_):** δ 21.13 (CH_3_), 30.07 (CH_3_), 49.98 (CH_2_), 55.85 (CH_3_), 67.07 (CH_2_), 111.03 (ArCH), 111.81 (ArCH), 121.29 (ArCH), 127.87 (ArC), 148.99 (ArC), 149.19 (ArC), 166.96 (C=O), 200.40 (C=O) ppm.

**Benzo[d][1,3]dioxol-5-ylmethyl 3-oxobutanoate (3c).** Colourless oil (91% yield). **^1^H NMR (CDCl_3_):** δ 2.16 (s, 3H), 3.39 (s, 2H), 4.98 (d, J = 2.7 Hz, 2H), 5.85 (m, 2H), 6.69 (d, J = 7.7 Hz, 1H), 6.74 (dd, J = 7.4, 8.6 Hz, 2H) ppm. **^13^C NMR (CDCl_3_):** δ 31.14 (CH_3_), 50.04 (CH_2_), 67.10 (CH_2_), 89.63, 109.13 (ArCH), 108.27 (ArCH), 122.51 (ArCH), 129.02 (ArC), 147.64 (ArC), 166.95 (C=O), 200.39 (C=O) ppm.

**4-Methoxy-3-methylbenzyl 3-oxobutanoate (3d).** Yellow oil (75% yield). **^1^H NMR (CDCl_3_):** δ 2.24 (s, 3H), 2.25 (s, 3H), 3.48 (s, 2H), 3.80 (s, 3H), 5.08 (s, 2H), 6.82 (d, *J* = 8.3 Hz, 1H), 7.15 (m, 2H) ppm. **^13^C NMR (CDCl_3_):** δ 15.99 (CH_3_), 30.11 (CH_3_), 49.81 (CH_2_), 55.35 (CH_3_), 66.95 (CH_2_), 109.82 (ArCH), 126.92 (ArC), 127.50 (ArCH), 131.34 (ArCH), 158.02 (ArC), 167.06 (ArC), 167.06 (C=O), 200.48 (C=O) ppm.

**3-Methoxybenzyl 3-oxobutanoate (3e).** Colourless oil (70% yield). **^1^H-NMR (CDCl3):** δ 2.26 (s, 3H), 3.48 (s, 2H), 3.84 (s, 3H), 5.08 (s, 2H), 6.79 (m, 1H), 6.86-6.87 (m, 2H), 7.18-7.20 (m, 1H) ppm **^13^C-NMR (CDCl_3_):** δ 16.11 (CH_3_), 30.11 (CH_3_), 50.00 (CH_2_), 55.59 (CH_3_), 67.06 (CH_2_), 109.64 (ArCH), 127.37 (ArC), 128.00 (ArCH), 131.06 (ArCH), 157.84 (ArC), 167.47 (C=O), 200.32 (C=O) ppm.

**4-Methoxybenzyl 3-oxobutanoate (3f).** Colourless oil (90% yield). **^1^H-NMR (CDCl_3_):** δ 2.13 (s, 3H), 3.39 (s, 2H), 3.76 (s, 3H), 3.78 (s, 3H), 5.01 (s, 2H), 6.75 (s, 1H), 6.82 (s, 2H) ppm. **^13^C-NMR (CDCl_3_):** δ 21.13 (CH_3_), 30.08 (CH_3_), 49.98 (CH_2_), 55.30 (CH_3_), 67.06 (CH_2_), 113.98 (ArCH), 127.87 (ArC), 130.46 (ArCH), 159.19 (ArC), 167.16 (C, C=O), 200.40 (C, C=O) ppm.

1. **Purity (HPLC) of active final compounds.**

| **Compound** | **UPLC Purity** |
| --- | --- |
| **5d** | 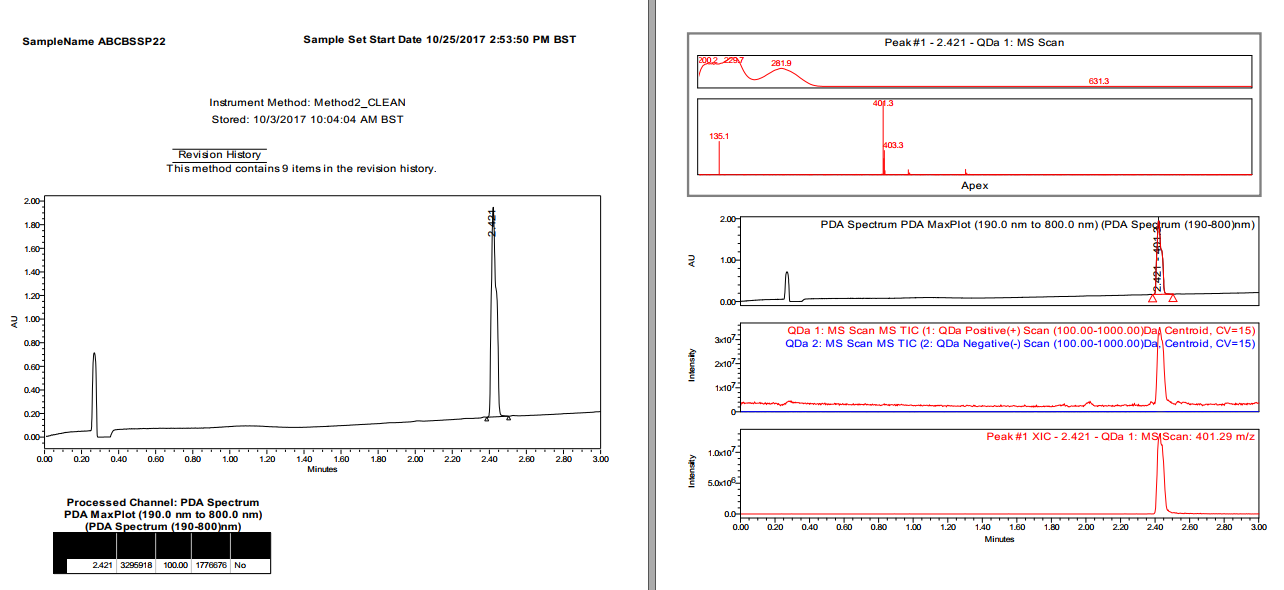 |
| **5e** | 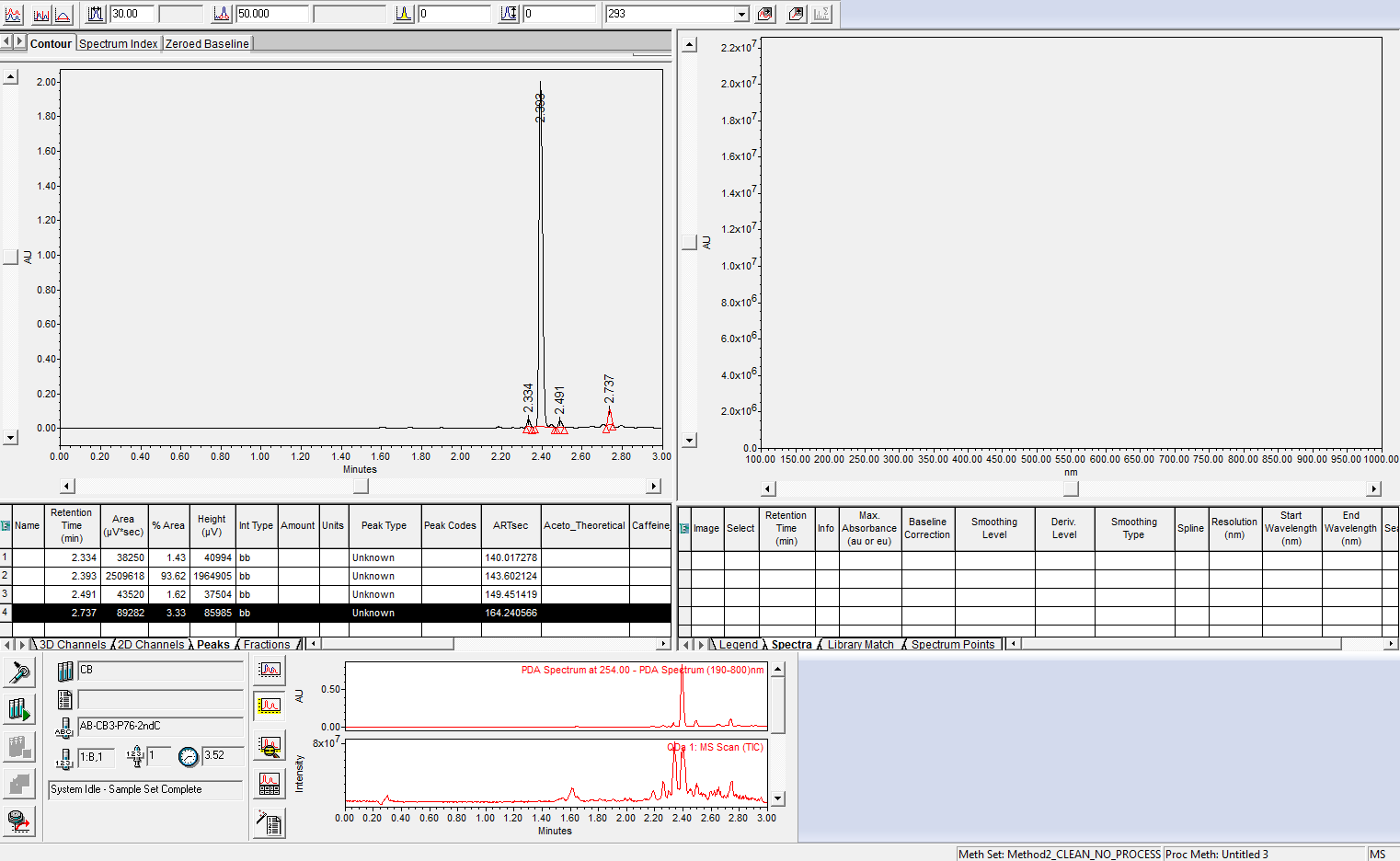 |
| **5f** | 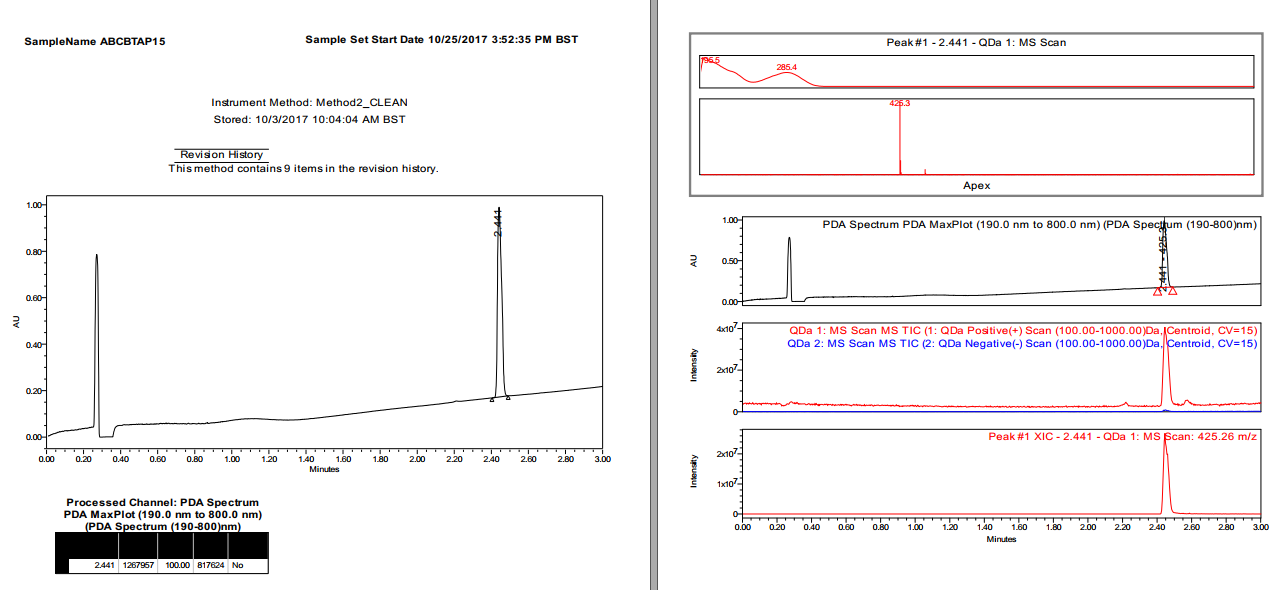 |
| **5g** | 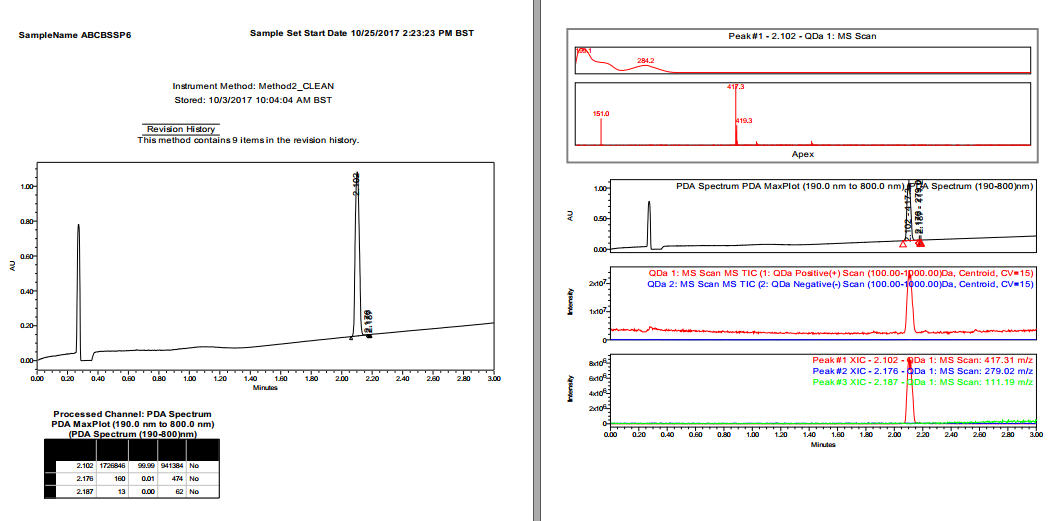 |
| **5h** | 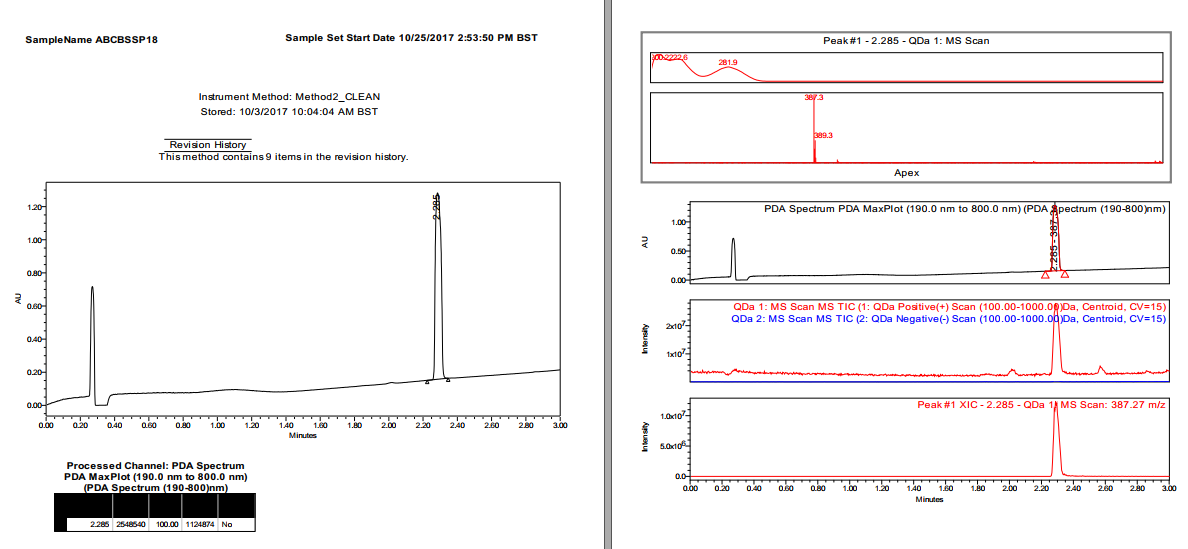 |
| **5i** | 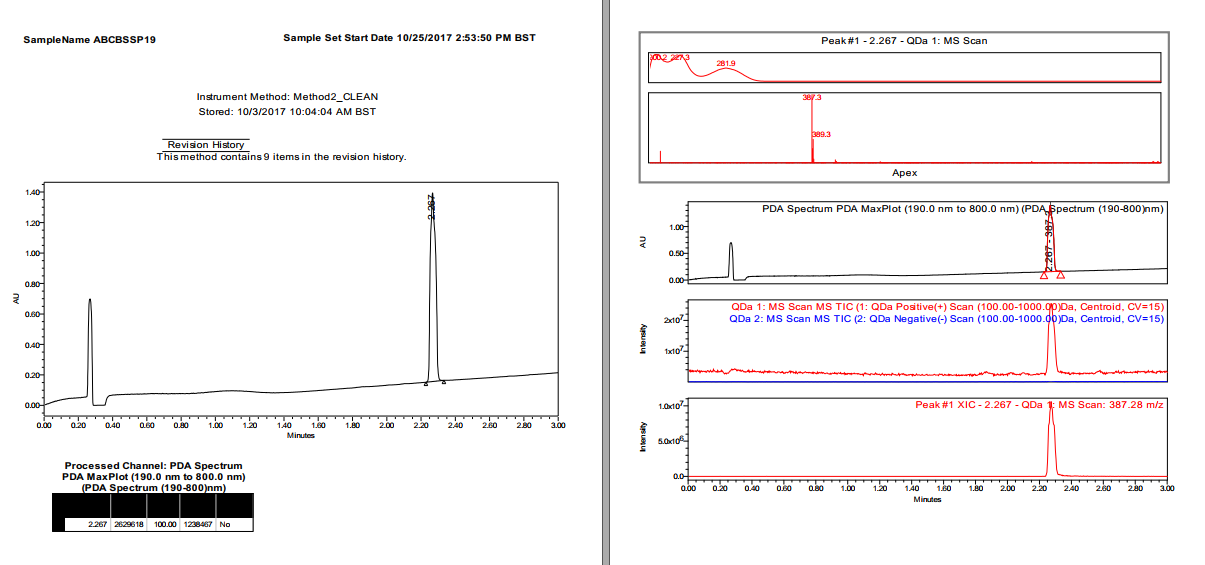 |
| **5j** | 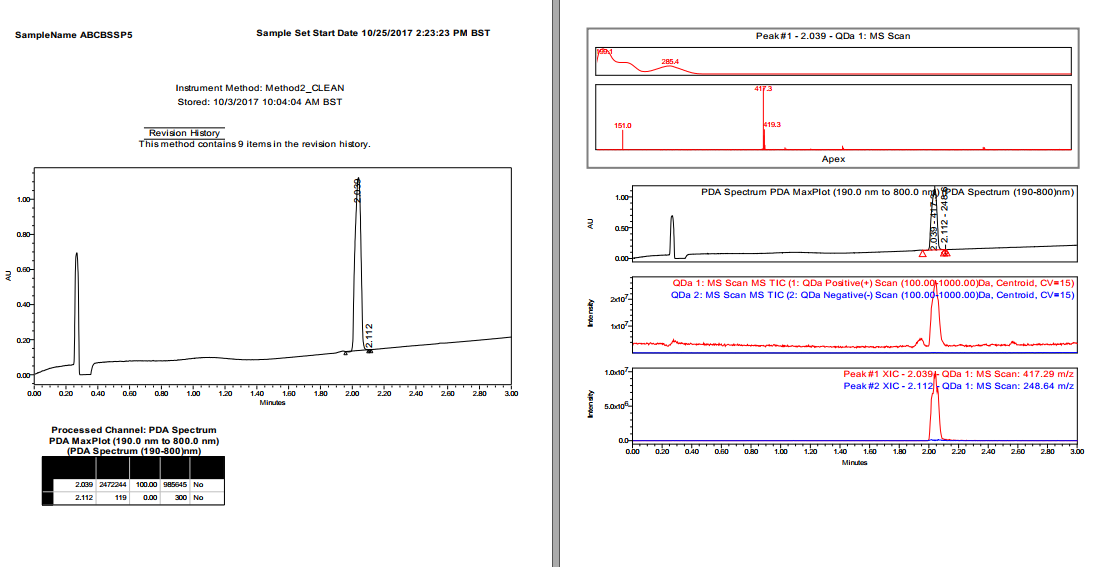 |

1. **NMR spectra (^1^H and ^13^C) of active final compounds**


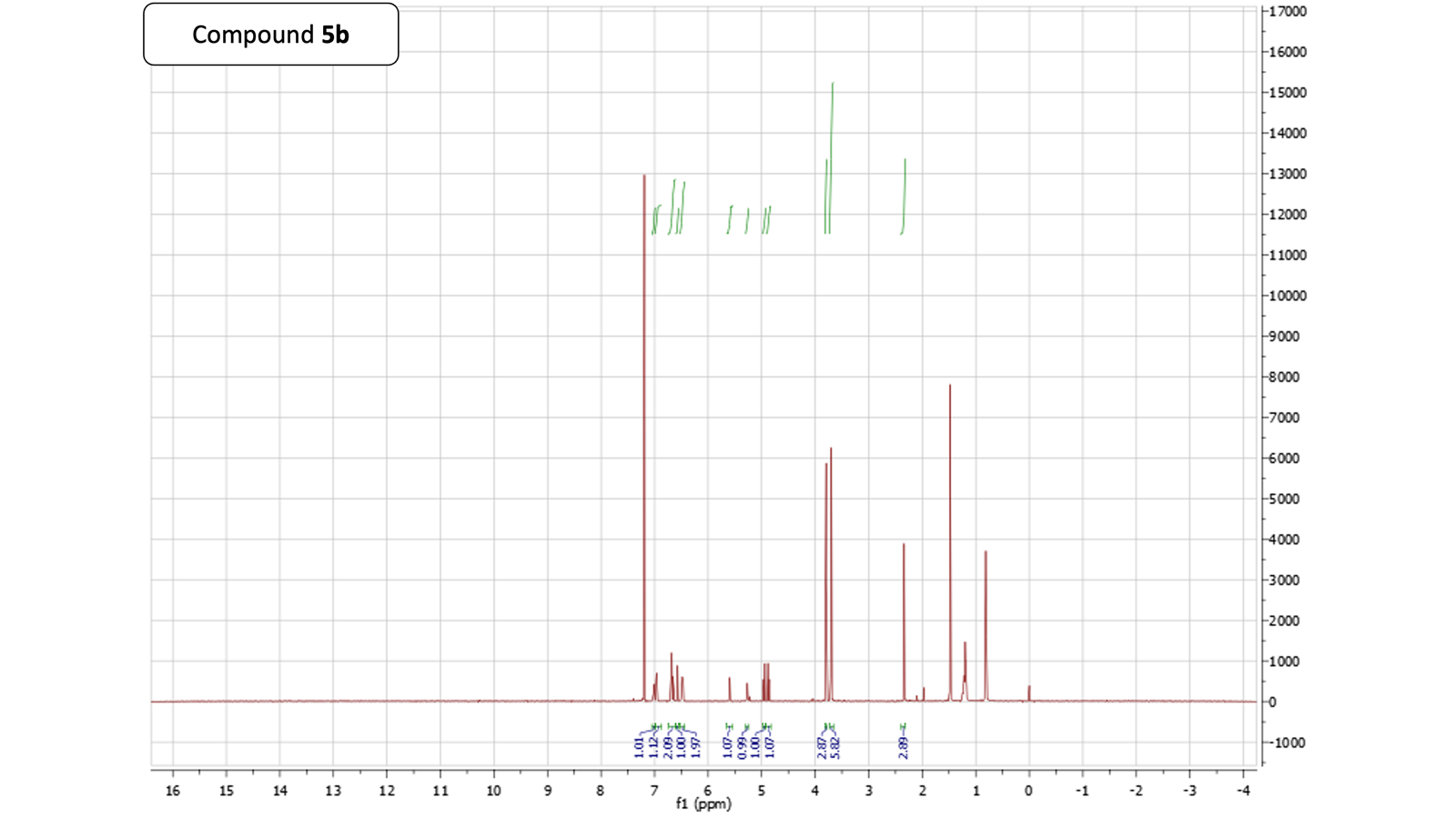


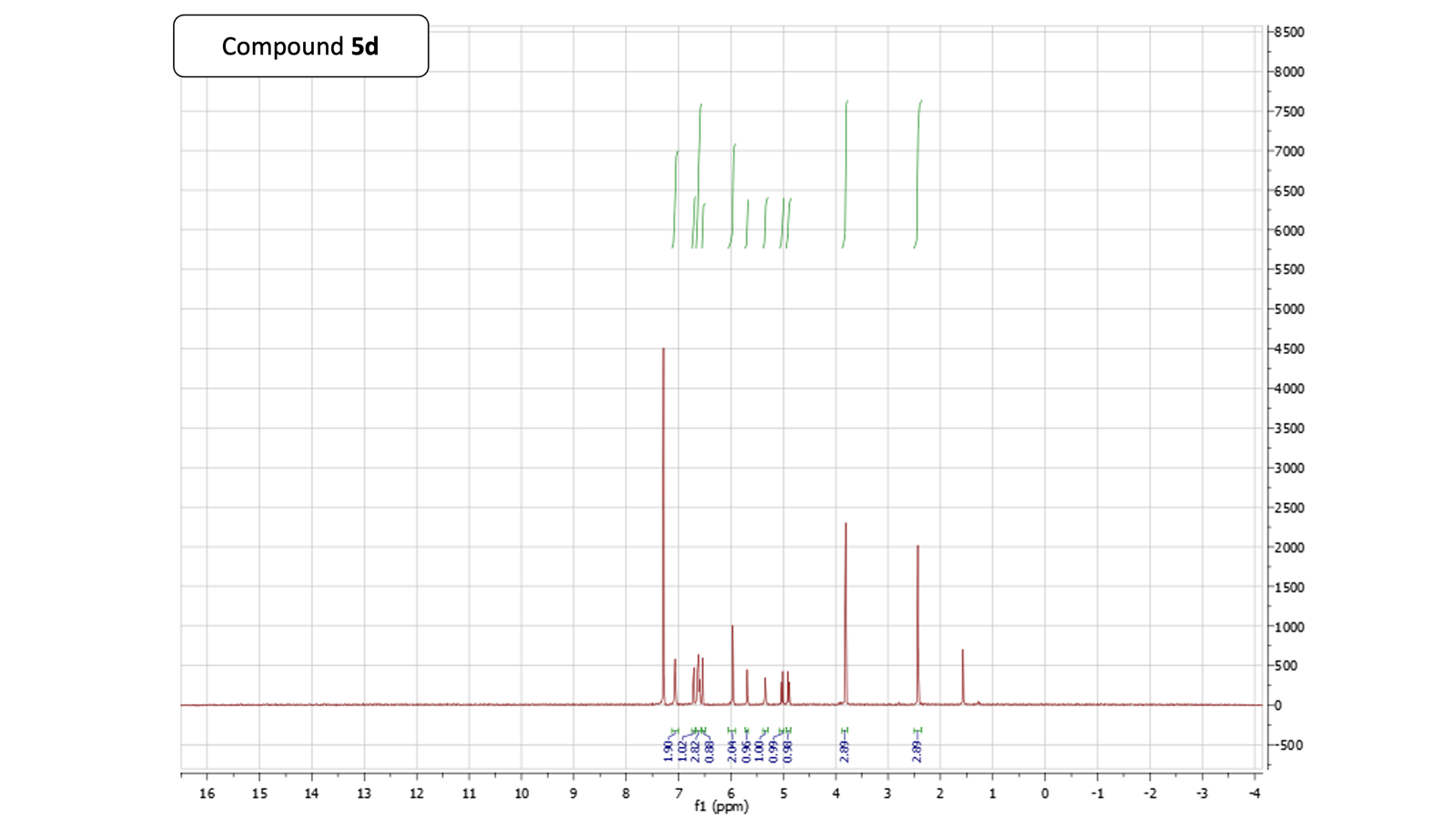


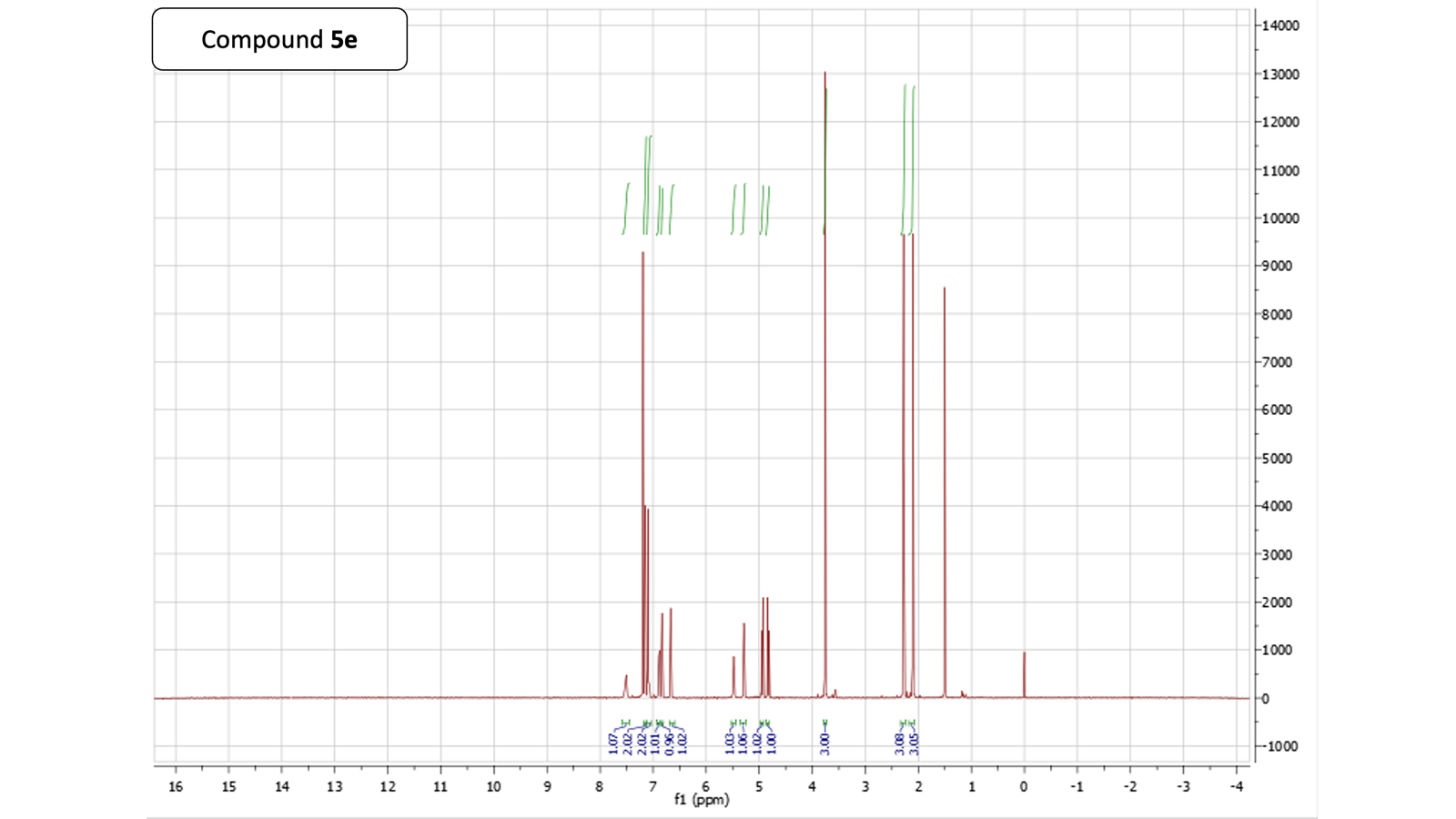


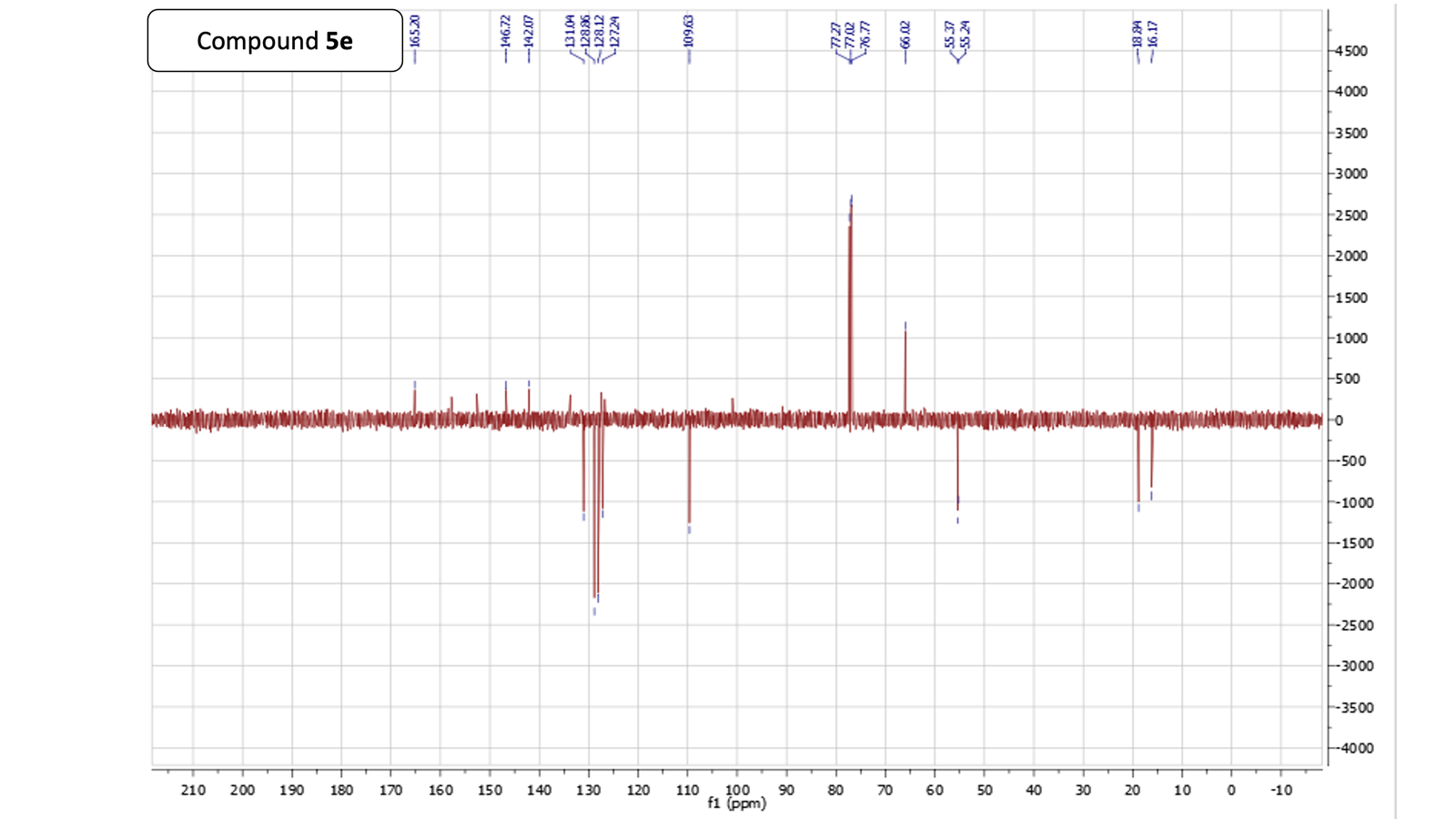


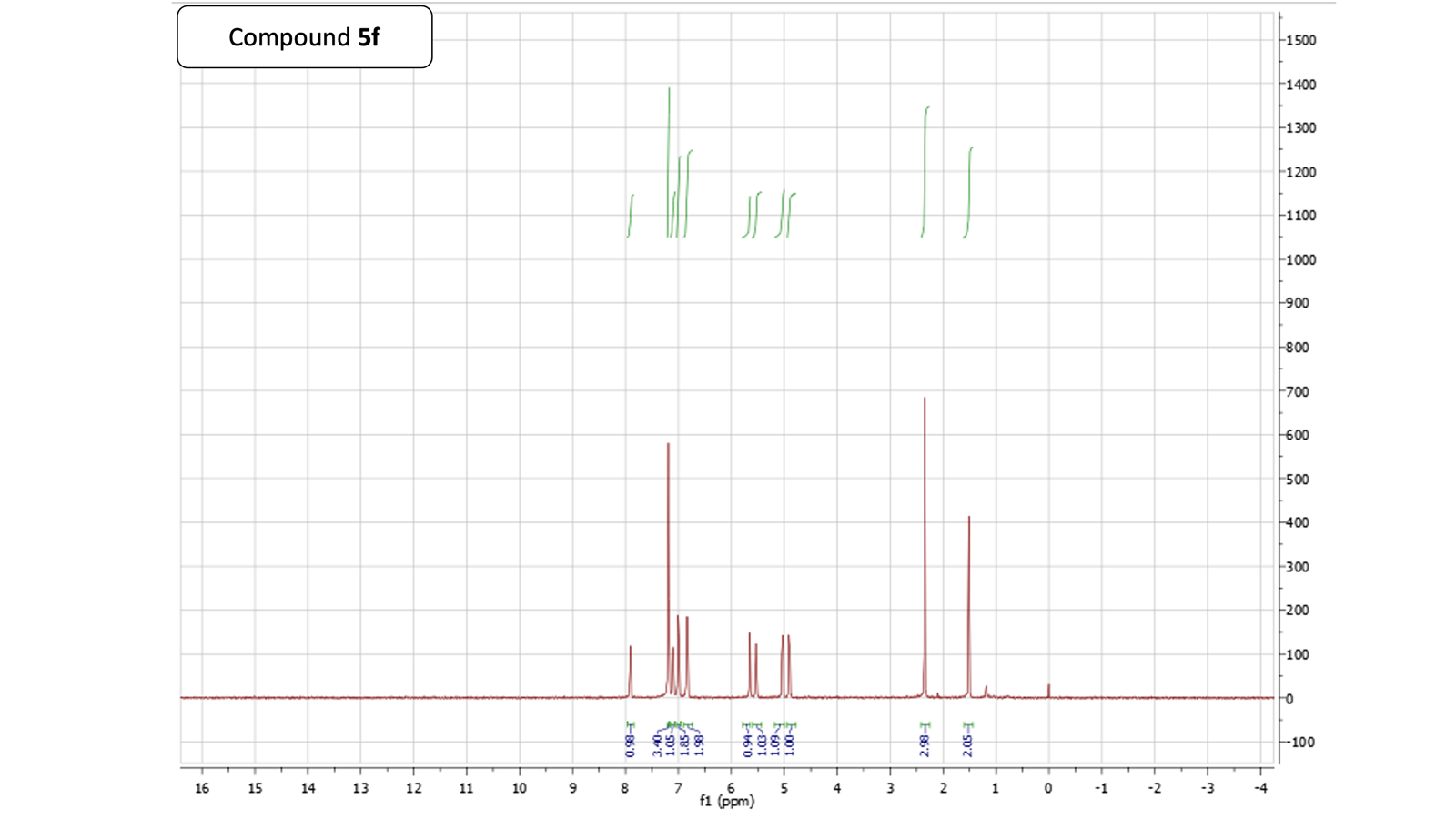


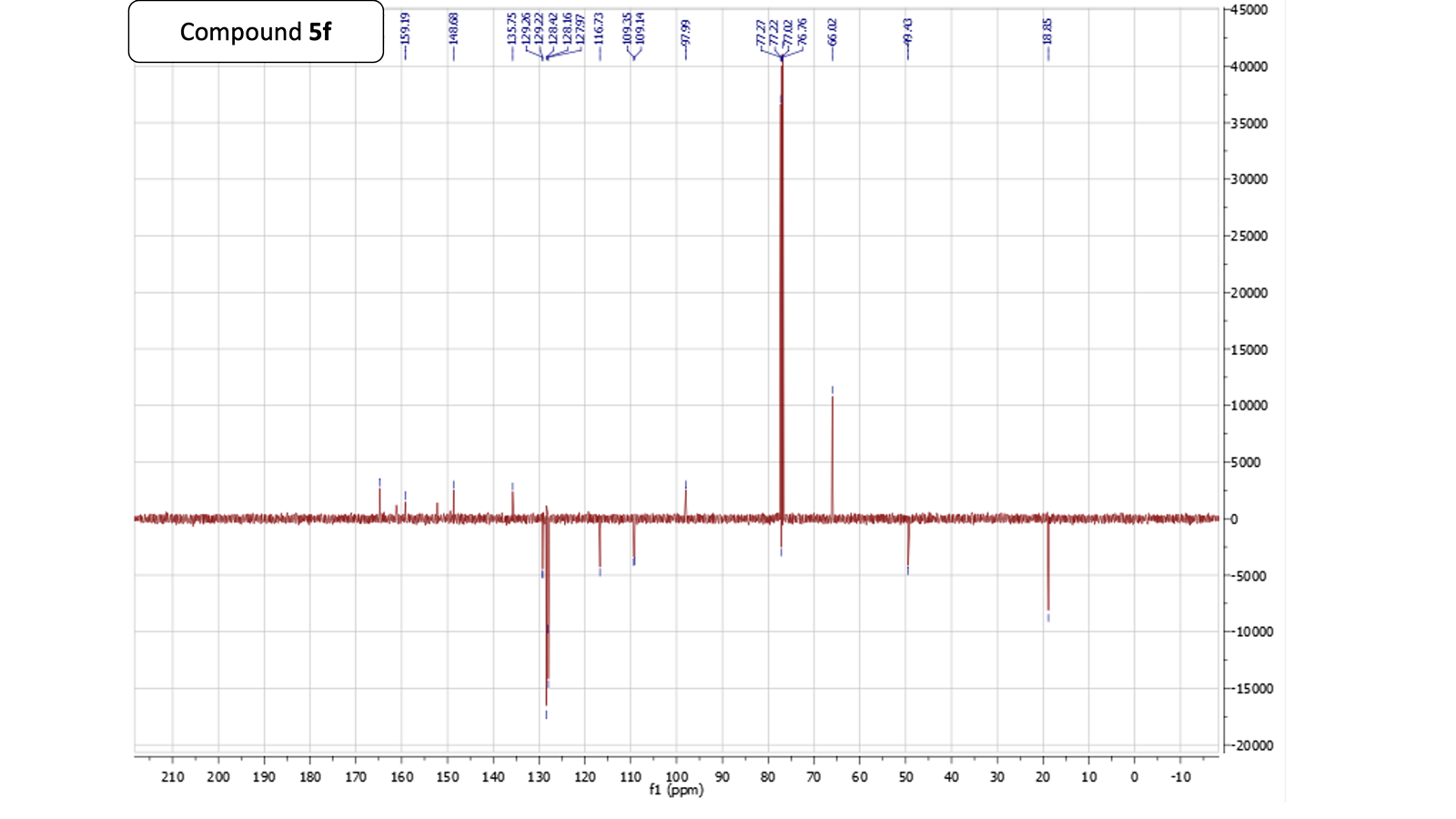


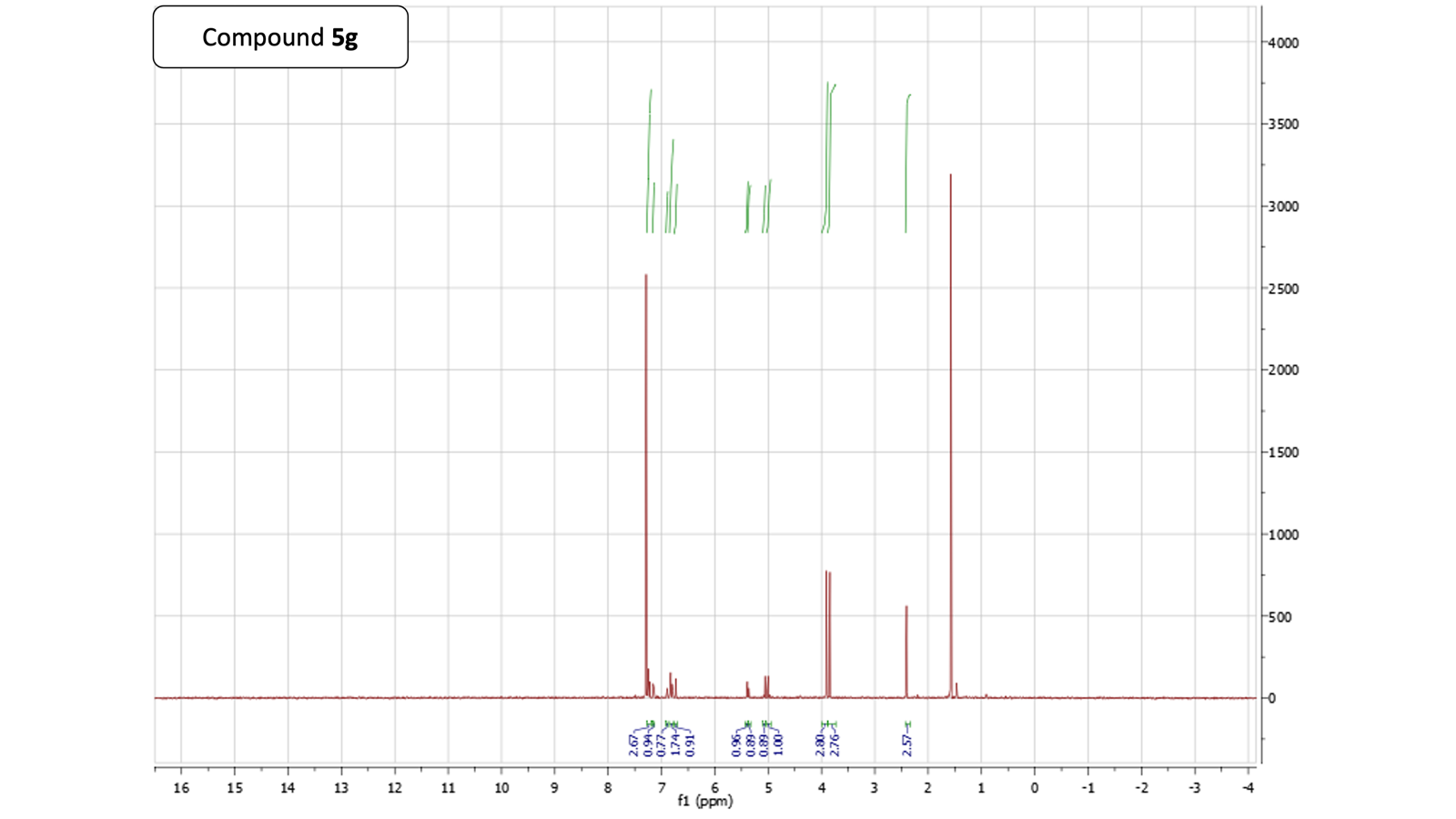


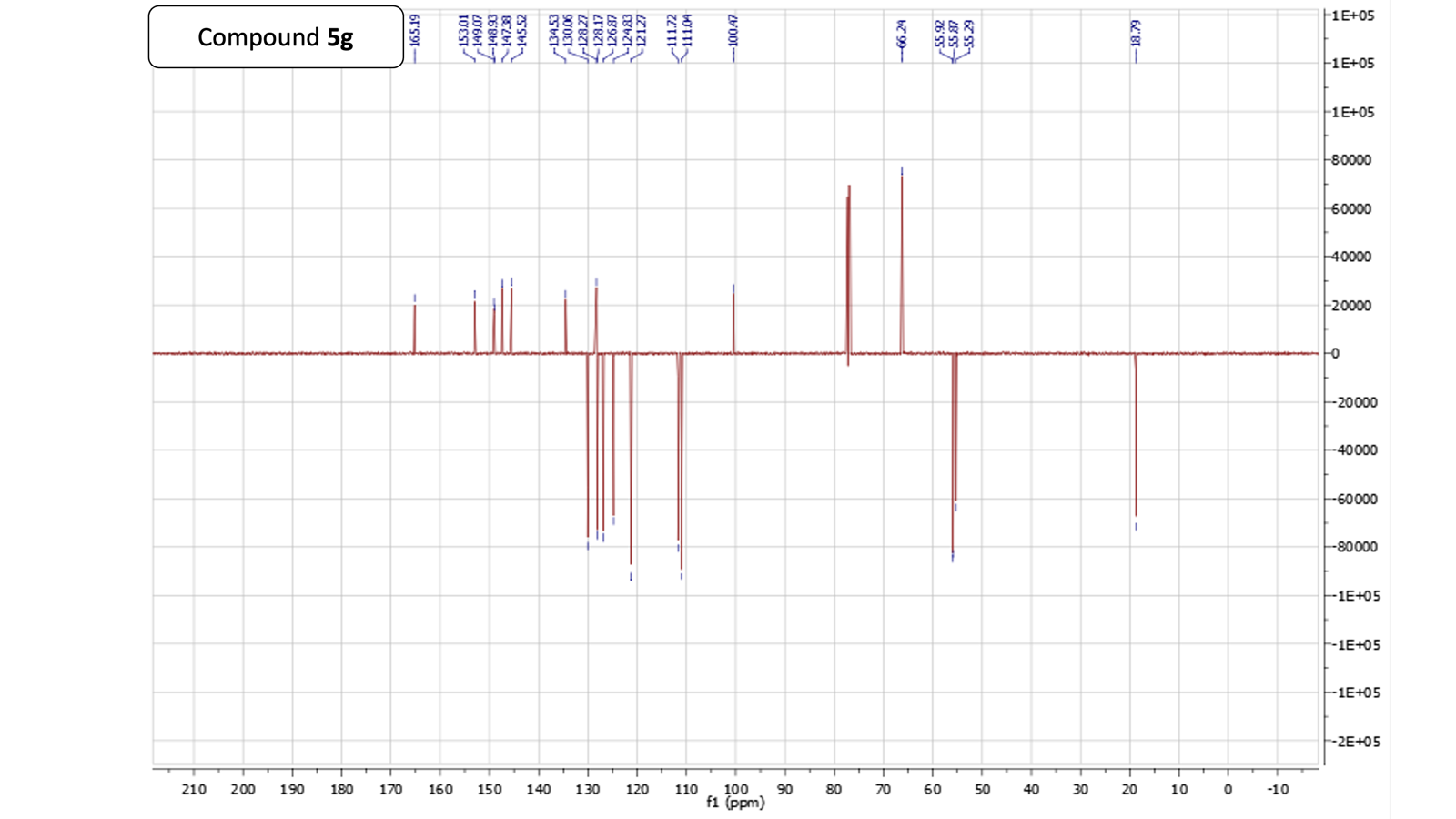


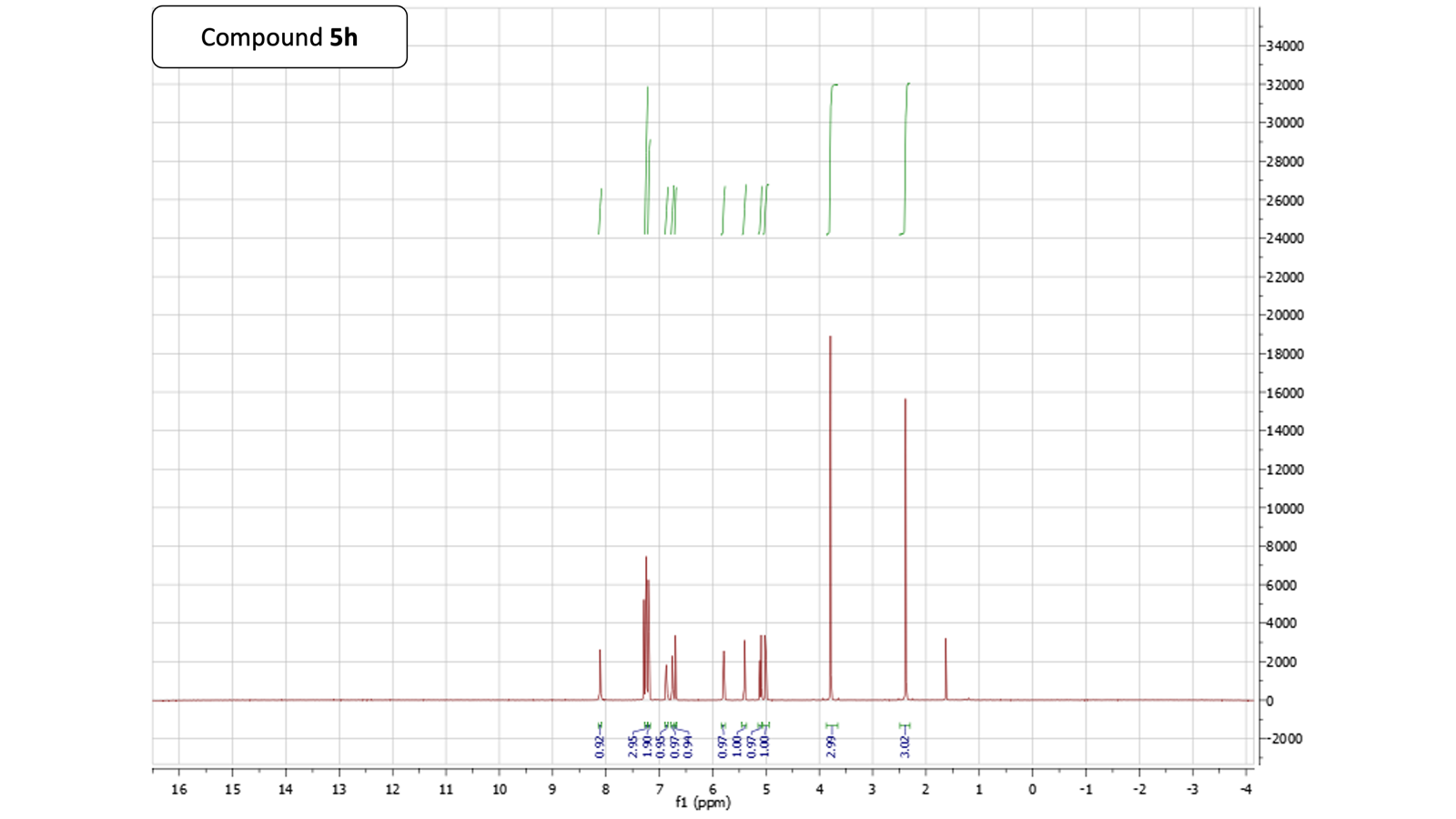


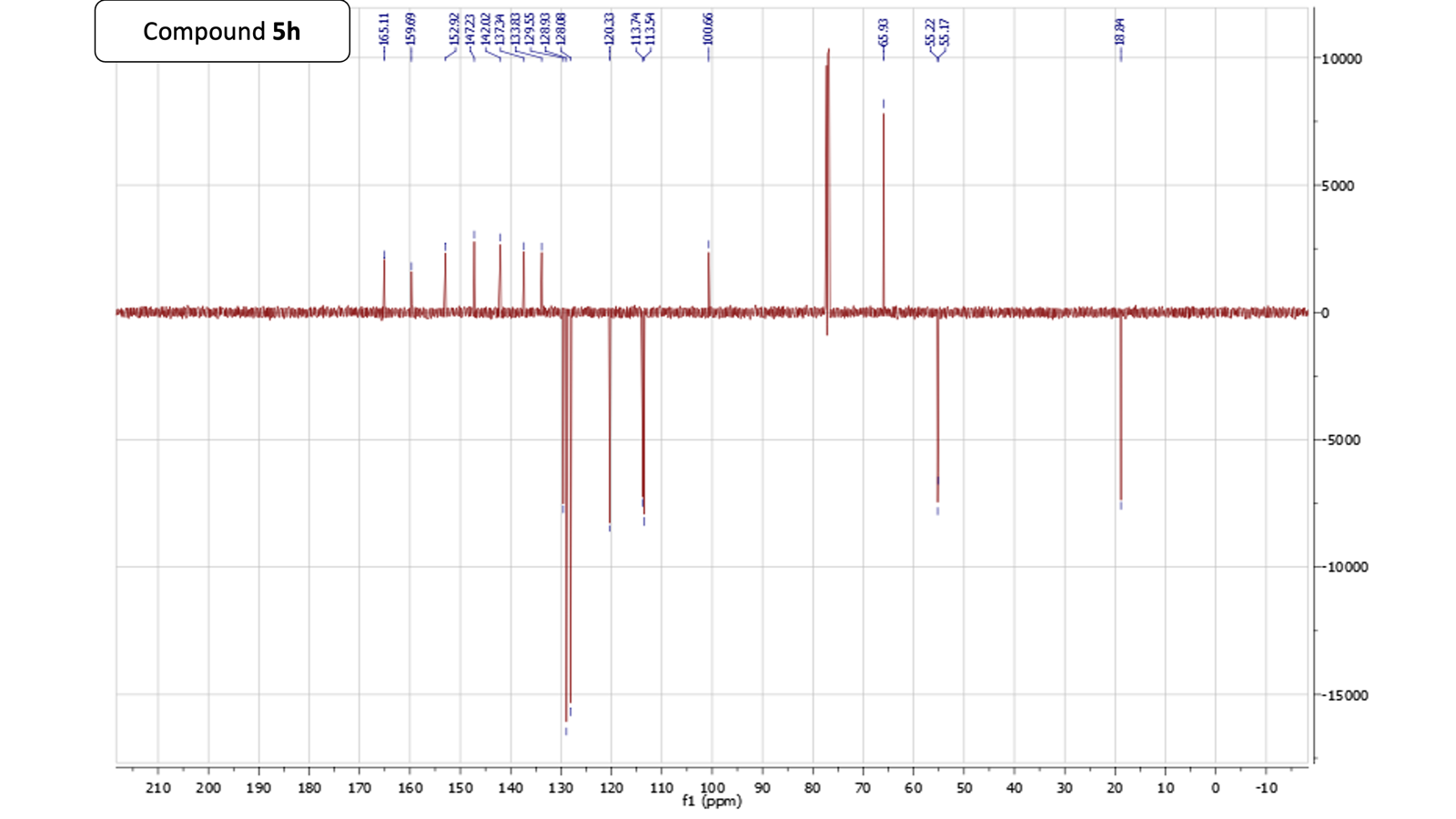


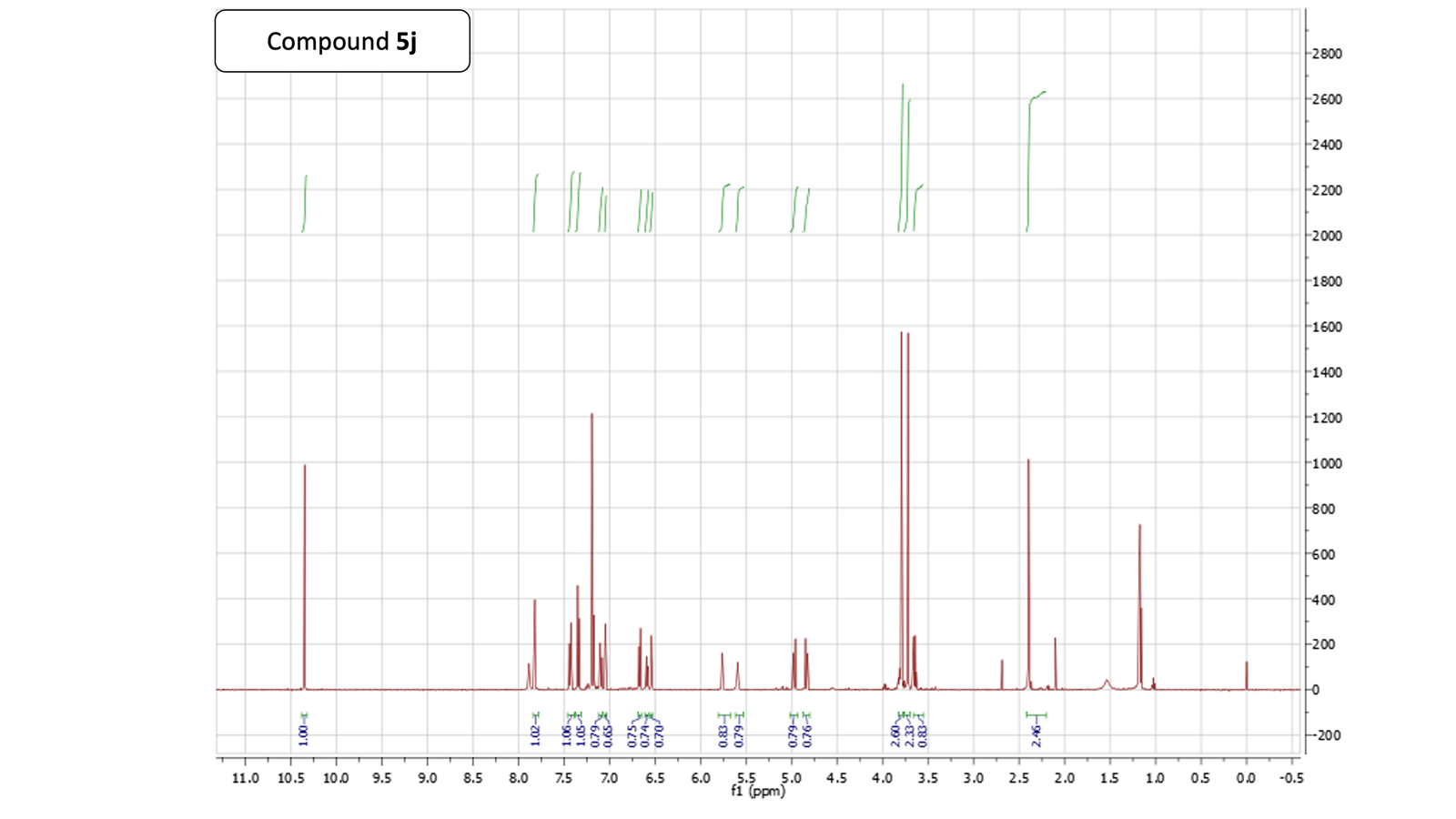


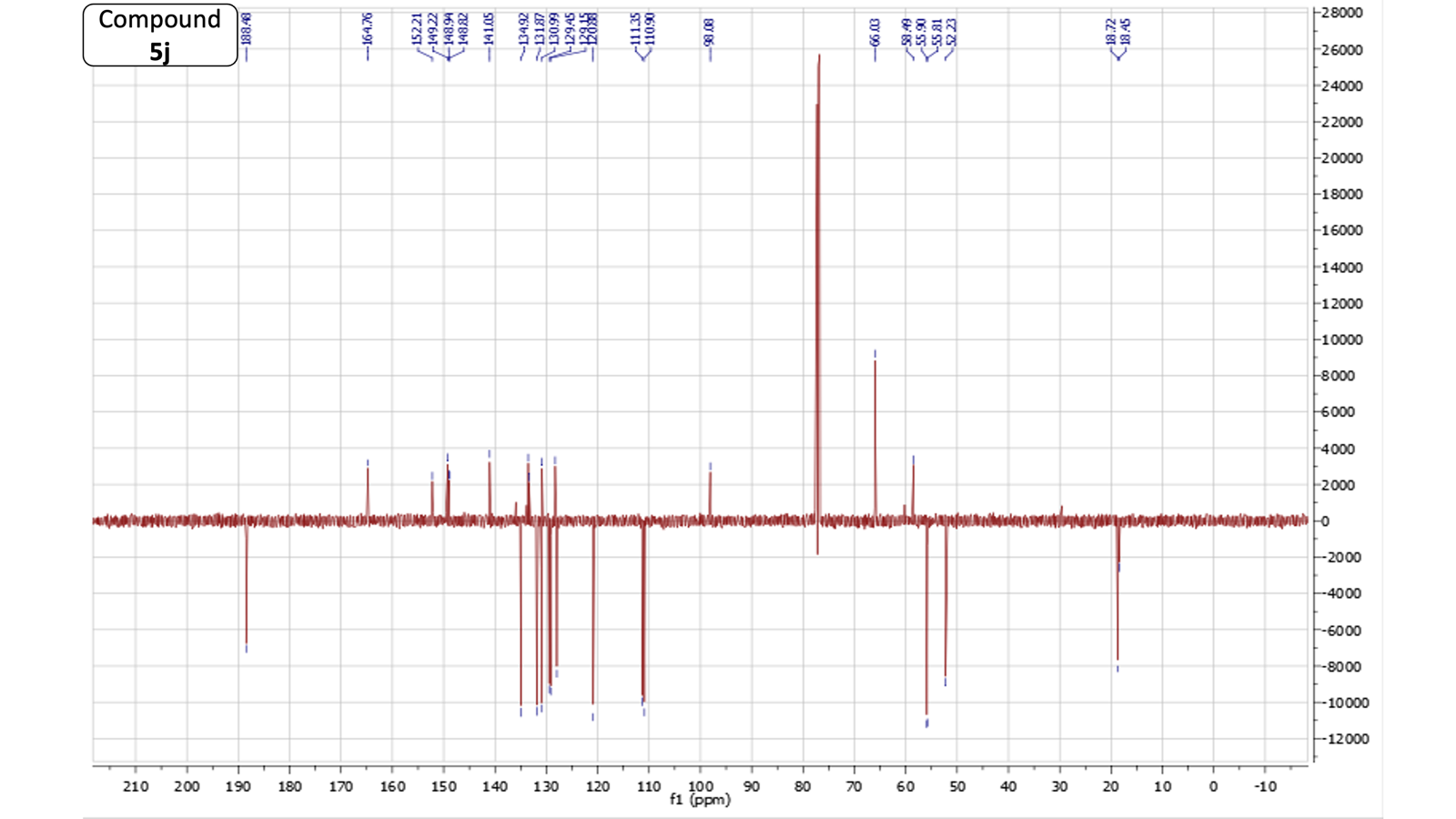


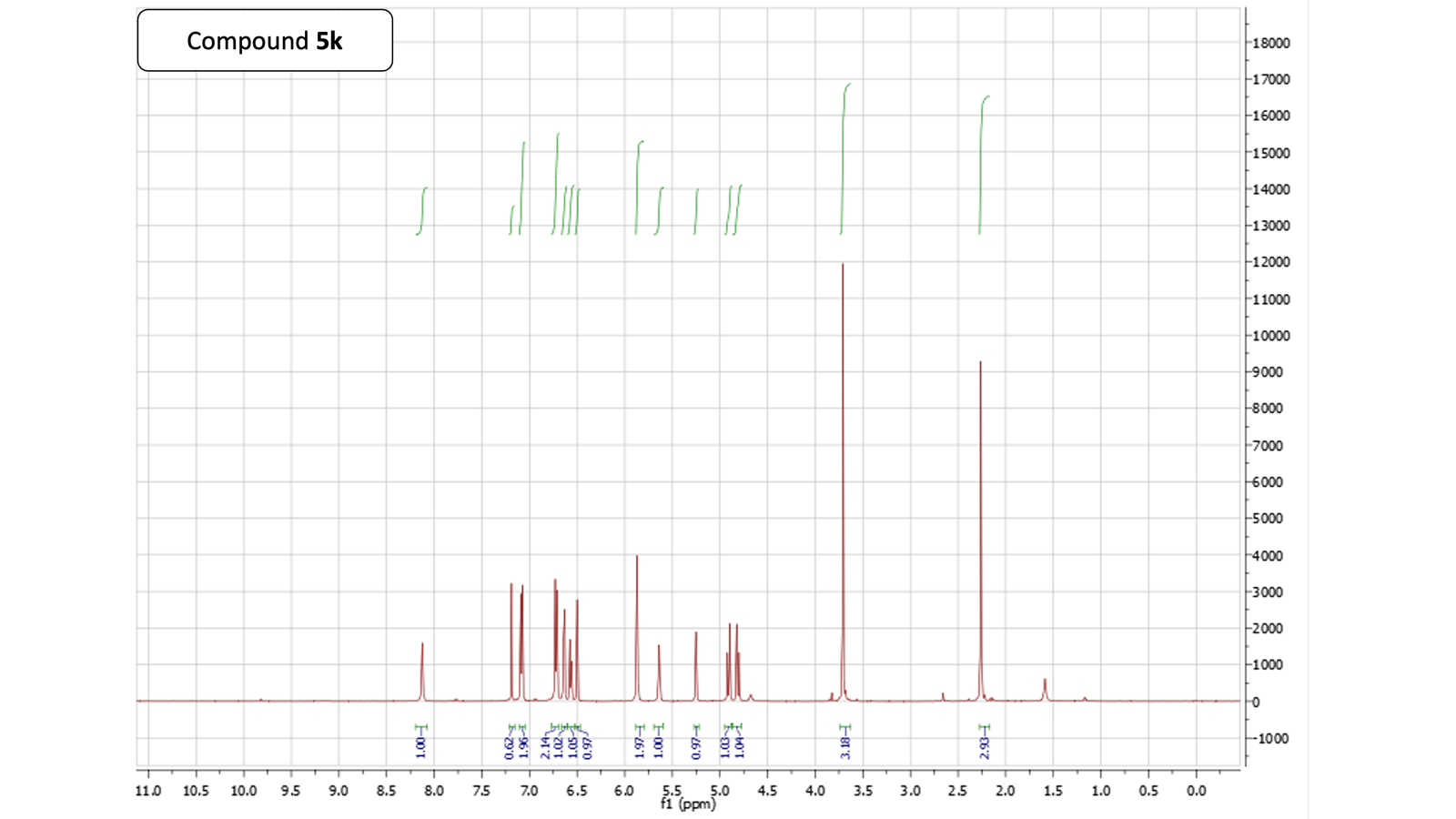


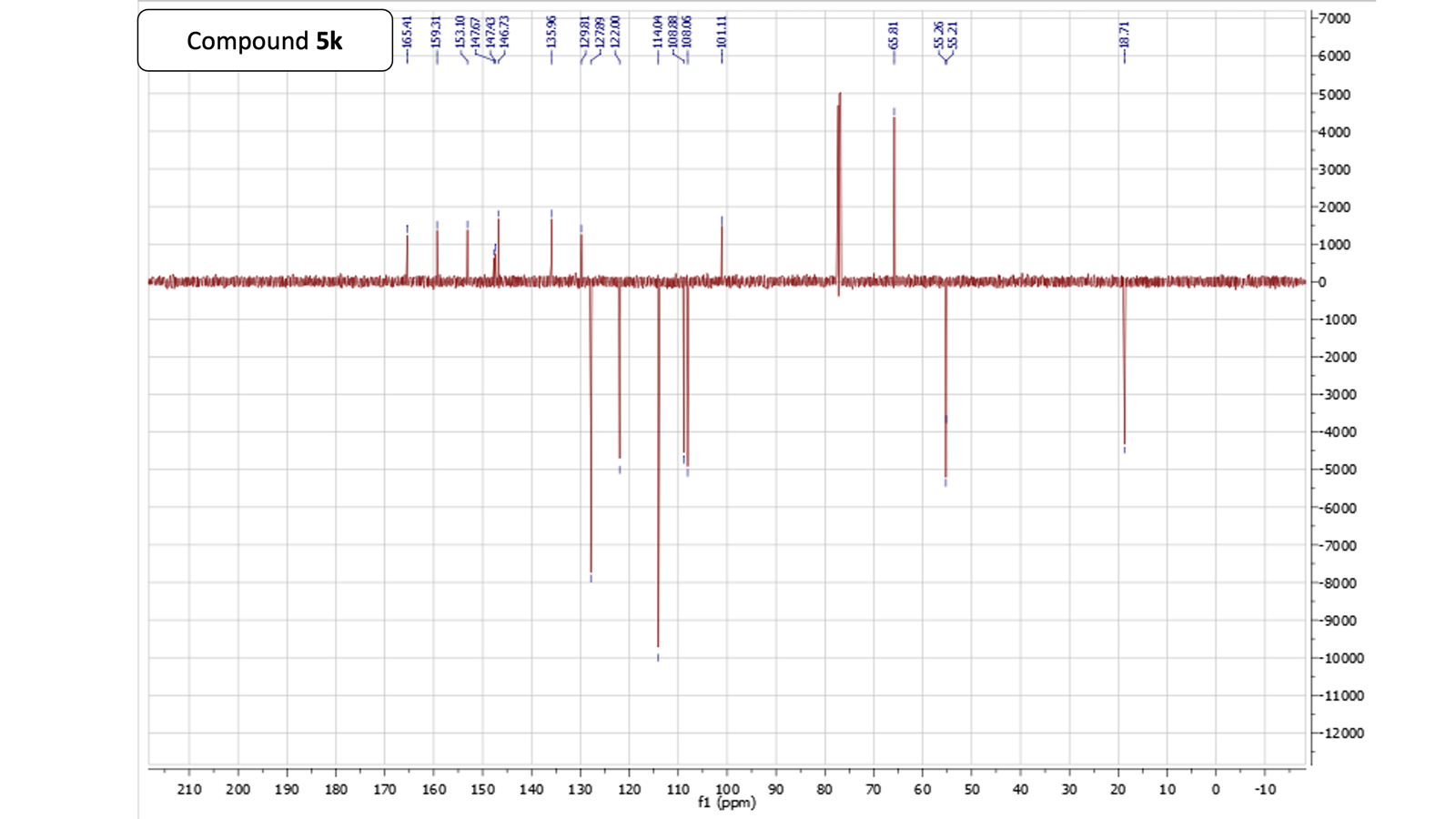


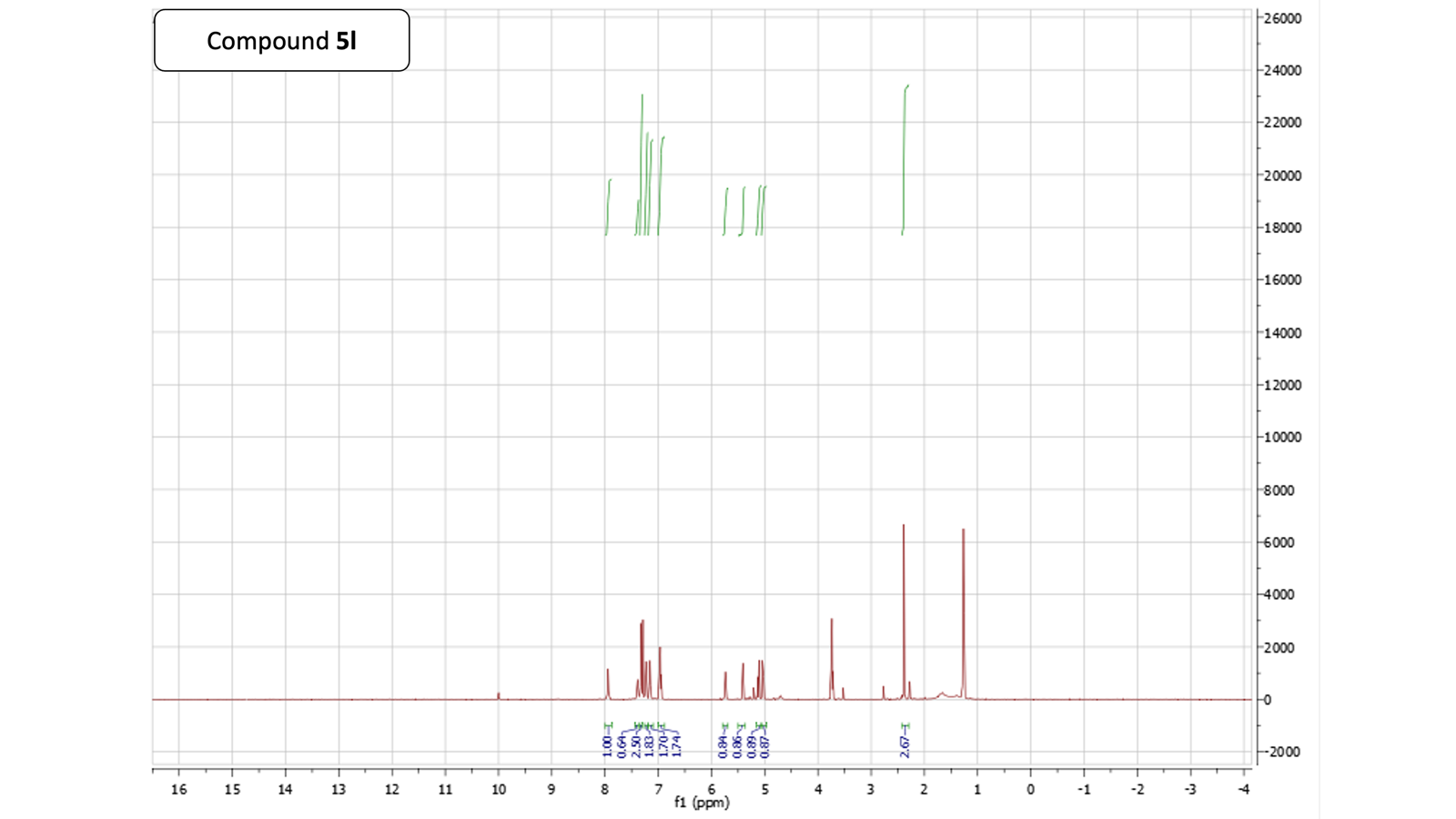


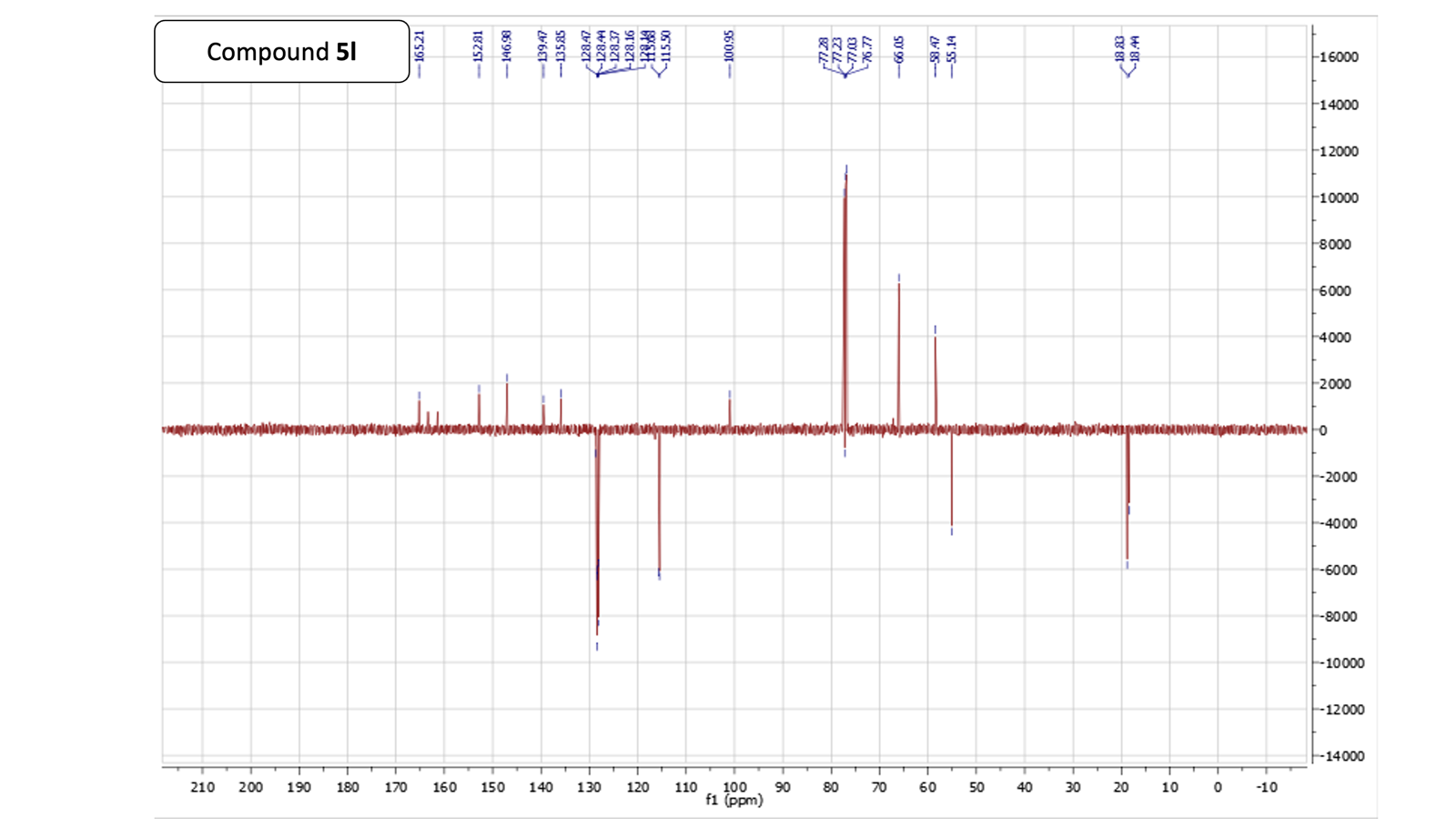


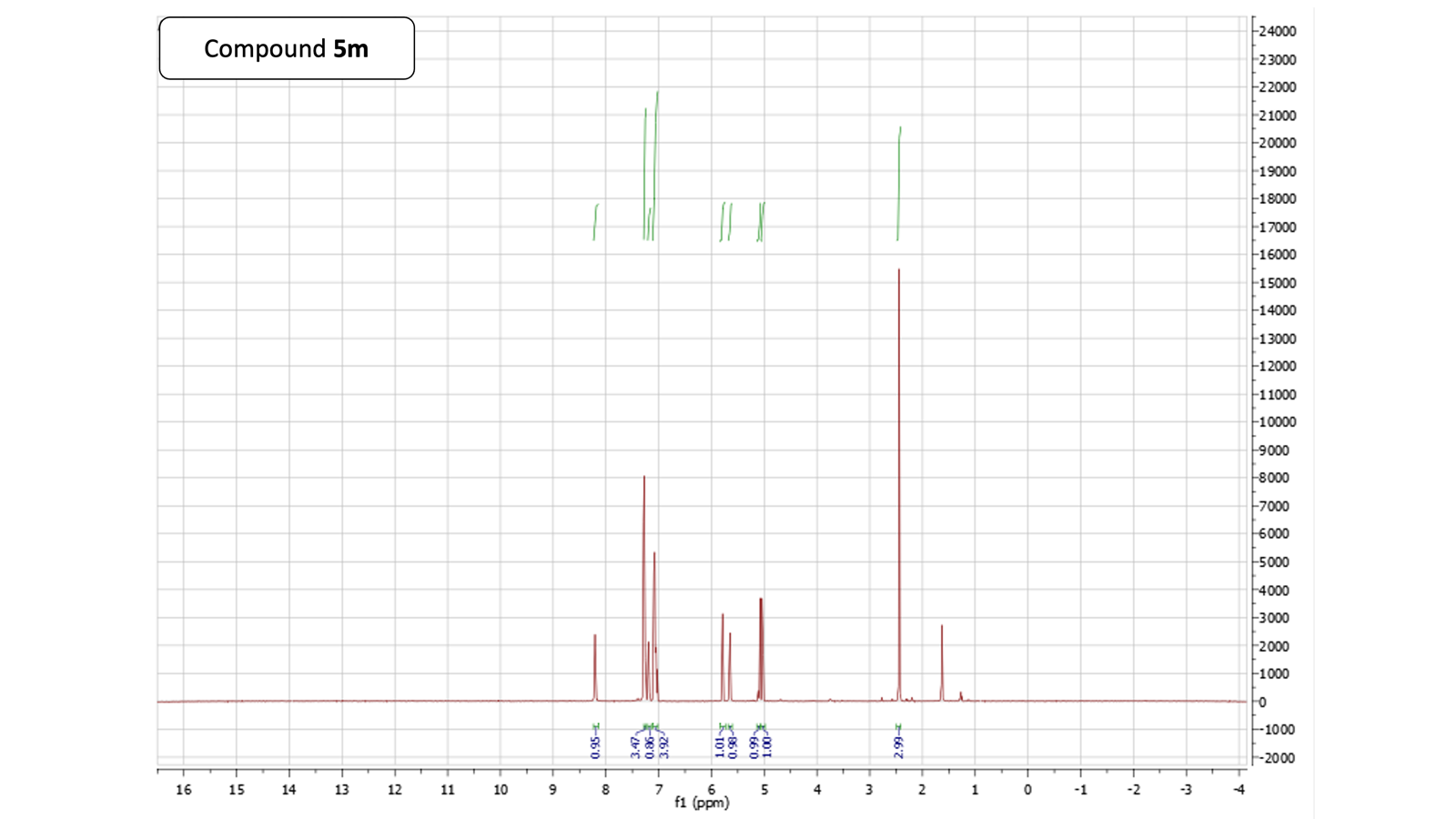


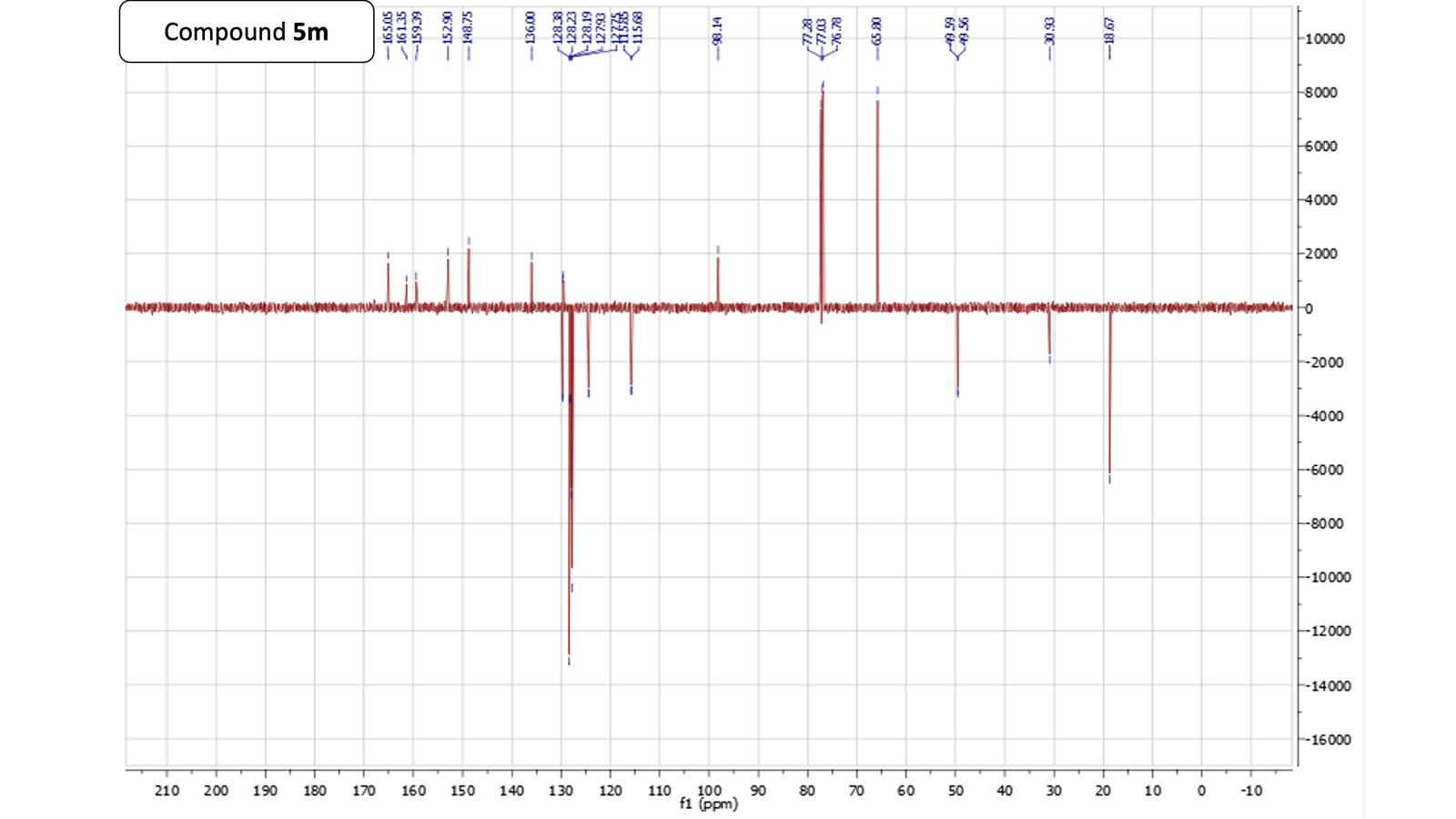


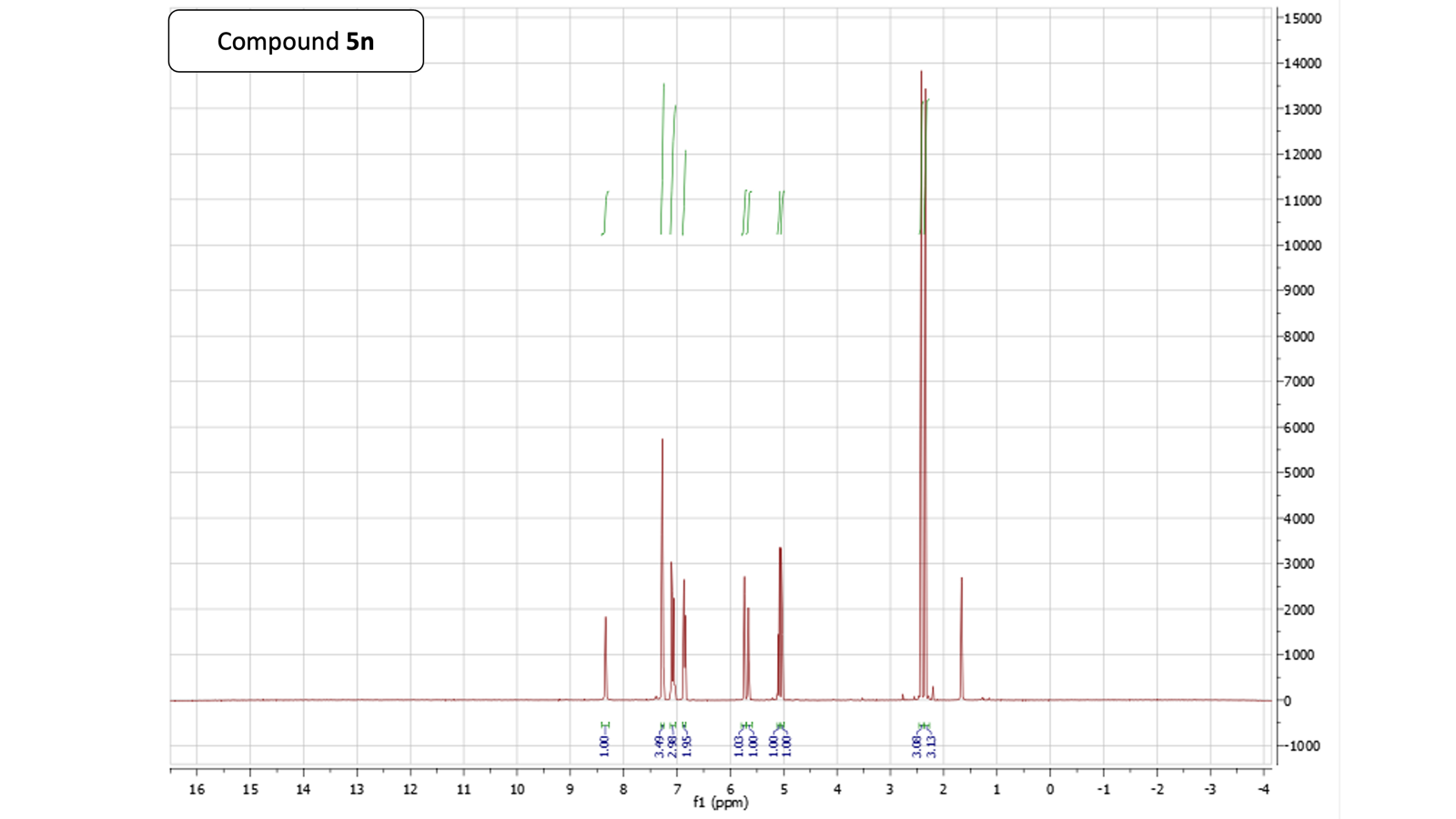


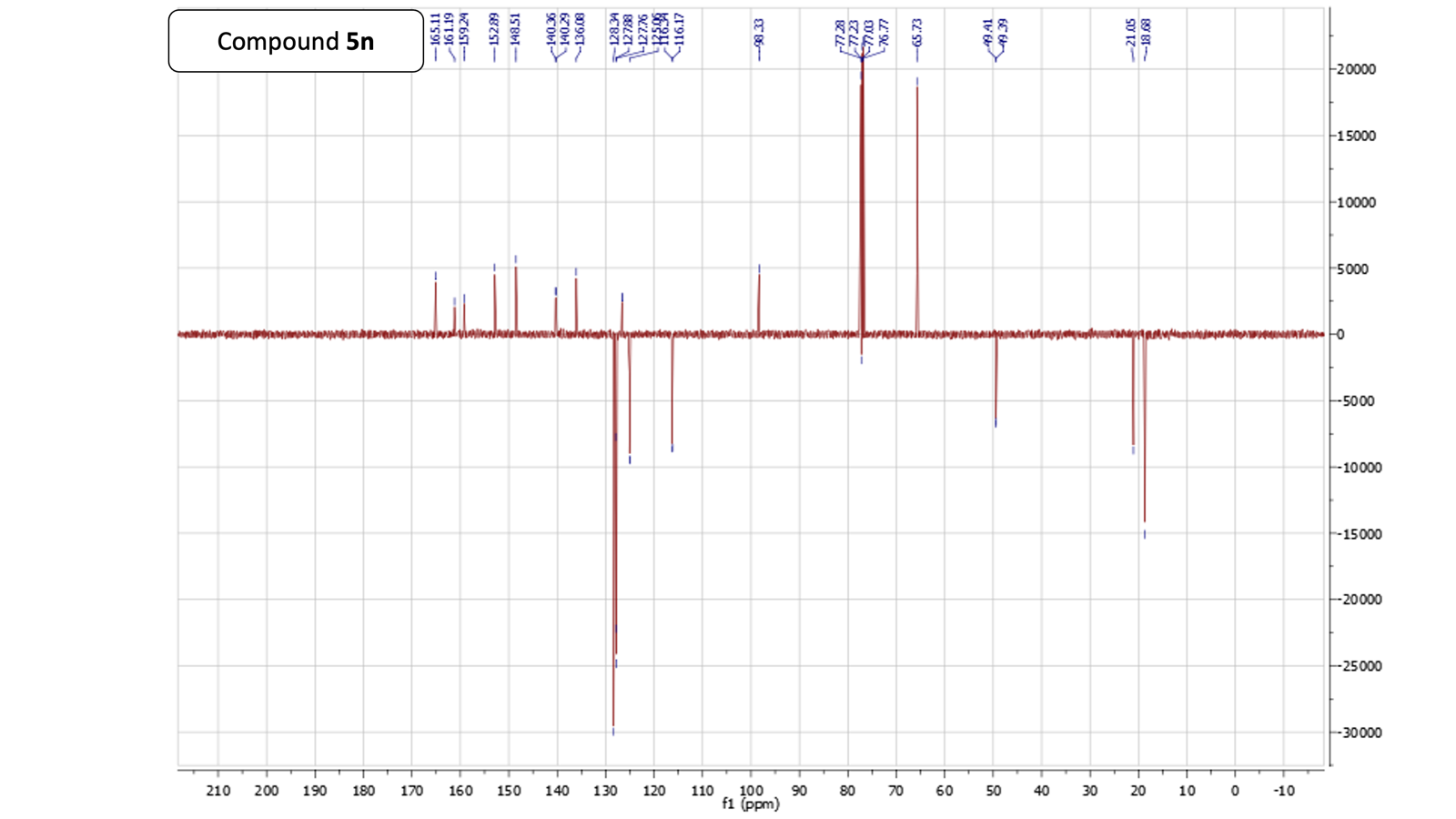

Supplement: Supplementary file 1 — Data S1: cbdd70221‐sup‐0001‐Supinfo1.docx. [file CBDD-106-e70221-s001.docx]
